# Immunogenicity and protective efficacy of recombinant BCG expressing a multi-epitope vaccine against mycobacterium tuberculosis

**DOI:** 10.3389/fcimb.2026.1912983

**Published:** 2026-09-30

**Authors:** Xuan-ji Zhang, Jia-rui Luo, Ting-ting Tian, Juan Shi, Zheng-long Chai, Chuang Li, Si-yu Zhang, Tao Qu, Feng-bo Zhang, Yue-jie Zhu

**Affiliations:** 1 Department of Clinical Laboratory, The First Affiliated Hospital of Xinjiang Medical University, Urumqi, Xinjiang, China; 2 Department of Clinical Laboratory, The Second People’s Hospital of Kashgar Prefecture, Kashgar, Xinjiang, China; 3 Reproductive Medicine Center, The First Affiliated Hospital of Xinjiang Medical University, Urumqi, Xinjiang, China

**Keywords:** ESAT-6.1, multi-epitope vaccine, recombinant BCG, shuttle plasmid, T7SS, tuberculosis (TB)

## Abstract

**Introduction:**

*Mycobacterium tuberculosis (Mtb)* infects approximately one-quarter of the global population, of whom 5%–10% develop active tuberculosis (TB). Although Bacillus Calmette–Guérin (BCG) remains the cornerstone vaccine against severe infant TB, its efficacy in adults is limited, underscoring the need for novel TB vaccines. This study aimed to design a multi-epitope vaccine, tMEV, and validate its protective potential through bioinformatic prediction and *in vivo* experiments.

**Methods:**

tMEV was constructed from dominant T- and B-cell epitopes derived from Mtb ESAT-6, PE35, PPE38, and PE4 proteins, fused with an adjuvant. Its vaccine candidacy was assessed via physicochemical and solubility profiling, secondary and tertiary structure modeling, TLR-4 molecular docking, molecular dynamics simulation, and immune simulation. The tMEV coding sequence was cloned into pMV361 and transformed into BCG to generate recombinant BCG (rBCG-tMEV). Immunogenicity, antigenicity, and protective efficacy were evaluated in mouse immunization and Mtb challenge models.

**Results:**

tMEV exhibited favorable vaccine-like properties *in silico*. In mice, both tMEV and rBCG-tMEV induced Mtb-specific immune responses. Compared with PBS, tMEV significantly enhanced cellular and humoral immunity with a Th2-biased profile. Relative to conventional BCG, rBCG-tMEV elicited a superior mixed Th1–Th2 response and higher antigen-specific immunity, accompanied by increased secretion of IFN-γ, IL-2, TNF-α, granzyme B, and perforin. Moreover, rBCG-tMEV markedly reduced bacterial loads in the lungs and spleens of Mtb-infected mice, conferring partial protection.

**Discussion:**

These findings demonstrate that tMEV and rBCG-tMEV possess robust immunogenicity and protective potential against Mtb, representing promising novel TB vaccine candidates and providing a rationale for future vaccine development and optimization.

## 1 Introduction

Tuberculosis (TB) is an infectious bacterial disease caused by Mycobacterium tuberculosis (Mtb). It is mainly transmitted through the respiratory tract, and people usually contract the disease by inhaling aerosol particles containing the bacteria (Naqvi et al., 2025). This pathogen primarily invades pulmonary tissue, causing pulmonary tuberculosis, but can also spread to other organs via the bloodstream or lymphatic system, leading to extrapulmonary tuberculosis conditions such as tuberculous meningitis (Spatola et al., 2024). As the COVID-19 pandemic gradually recedes, tuberculosis has re-emerged as one of the leading causes of human deaths attributable to a single infectious pathogen (Dheda et al., 2022; Falzon et al., 2023; World Health Organization, 2025). Against the backdrop of the global fight against tuberculosis, a study predicts that if the current situation remains unchanged, the positivity rate of immune responses to Mycobacterium tuberculosis in countries including China, India, the Philippines and Vietnam will keep declining by 2050 (MaChado et al., 2026). Meanwhile, asymptomatic tuberculosis is becoming increasingly prevalent, posing challenges to traditional symptom-based screening strategies (Mulenga et al., 2025). The Bacillus Calmette-Guérin (BCG) vaccine, derived from Mycobacterium bovis, is currently the only vaccine approved worldwide for the prevention of tuberculosis (Palmer et al., 2025). However, as a live attenuated vaccine, its efficacy in adults remains controversial (Darrah et al., 2020). To effectively address the challenges posed by TB, there is an urgent need to accelerate the development and deployment of innovative prevention and control strategies, as well as new vaccines.

Traditionally, vaccines have been produced using live or attenuated microorganisms, or components thereof. However, the redundant biological components or biological impurities present in such vaccines may trigger non-specific immune activation and adverse immune reactions (Jiang et al., 2023). Peptide vaccines offer an attractive alternative strategy, relying on short peptide fragments to induce highly targeted immune responses while avoiding adverse immune reactions (Mubarak et al., 2024). In addition, cellular immunity against Mtb has been shown to be more effective in protecting the host from TB infection, and epitope-based vaccines are characterized by specificity and precision. Therefore, epitopes capable of stimulating CD4^+^ and CD8^+^ T cells may represent ideal candidates for peptide-based vaccine design (Kumar et al., [[NoYear]]).

The type VII secretion system (T7SS) of Mycobacterium tuberculosis (Mtb) constitutes the core of its pathogenicity, not only responsible for secreting critical virulence effector proteins but also profoundly influencing the establishment of host-pathogen interactions, making it a highly promising therapeutic target for tuberculosis treatment (Tiwari et al., [[NoYear]]; Famelis et al., 2019). As the most potent T-cell antigen secreted by Mtb, early secreted antigen target 6 (ESAT-6) plays multiple roles in regulating the host’s immune response to infection, including inhibiting host T cells from producing IFN-γ, IL-17, and TNF-α, as well as inducing cell lysis (Sundaram et al., [[NoYear]]). In addition, ESAT-6 may contribute to the regulation of T7SS assembly and function and is regarded as a potential vaccine candidate (Cao et al., 2016).

PE/PPE family proteins are not only highly immunogenic but may also facilitate immune evasion by Mtb through mechanisms such as interfering with phagosome maturation and disrupting phagosome–lysosome fusion (Ehtram et al., 2025). Among them, the conserved N-terminal Pro-Glu (PE) domain of PE35 (Rv3872) interacts with PPE family proteins, such as PPE68, and participates in the assembly of the ESX-1 secretion system and the secretion of effector proteins, including ESAT-6 and CFP-10 (Zriba et al., [[NoYear]]). Studies have shown that deletion of PE35 leads to abnormal intracellular accumulation of ESAT-6 and CFP-10, significantly impairing the survival of Mycobacterium tuberculosis within macrophages (Brodin et al., 2006). PE35 has been recognized as a promising vaccine candidate (Gcebe et al., 2016). PE4, a protein secreted by ESX-3, is involved in iron acquisition and in overcoming host-induced iron restriction (Sundaram et al., [[NoYear]]). Previous studies have shown that PE4 expression is increased during Mtb infection and that PE4 is recognized as an immunodominant antigen capable of eliciting a strong humoral response in patients (Singh et al., 2012). The ESX-5 substrates PPE-MPTR and PE_PGRS comprise more than 80 proteins and are affected by mutations in PPE38, leading to altered secretion and reduced pro-inflammatory responses in infected macrophages. PPE38-mediated secretion of ESX-5 substrates is one of the core mechanisms by which pathogens achieve immune evasion and virulence attenuation through regulating host inflammatory responses (Ates et al., 2018). These findings suggest that secreted proteins from the T7SS of different Mtb strains may serve as important candidates for epitope vaccine development.

To maintain sufficient levels of antibody-mediated and cellular immunity against pathogens and to prevent potential TB infection, this study aimed to develop a multi-epitope recombinant BCG vaccine based on the ESAT-6, PE35, PPE38, and PE4 proteins of Mtb. Through coordinated multi-system antigenic responses, this strategy may reduce the likelihood of Mtb immune evasion, enhance host immune responses, and minimize potential adverse effects associated with candidate vaccine proteins.

The multi-epitope vaccine (tMEV) is a novel vaccine constructed by identifying highly antigenic cytotoxic T lymphocyte epitopes (CTLEs), helper T lymphocyte epitopes (HTLEs), linear B-cell epitopes (LBEs), and conformational B-cell epitopes (CBEs), with favorable hydrophilicity, stability, and non-toxicity. An immunoadjuvant sequence was subsequently added to enhance antigenicity, and a His-tag sequence was incorporated for protein purification. Multiple analyses, including secondary and tertiary structure modeling, optimization, and quality assessment, were performed using bioinformatics tools to ensure the stability and reliability of the tMEV protein. Molecular docking and molecular dynamics simulations were conducted to evaluate the affinity and interaction between tMEV and TLR-4. Finally, immune simulation was performed to model immune response types and dynamic changes *in vivo*. Predictive bioinformatics analysis indicated that the multi-epitope vaccine tMEV has potential as a vaccine candidate. tMEV was synthesized and integrated into the plasmid vector pMV361. The recombinant plasmid was then introduced into competent BCG to construct the rBCG-tMEV vaccine. The immunological and protective effects of tMEV and rBCG-tMEV were evaluated, and the results showed that both vaccines induced robust and specific cellular and humoral immune responses. Notably, rBCG-tMEV reduced Mtb colonization in the spleens and lungs of mice.

## 2 Materials and methods

### 2.1 Bioinformatics prediction methods

#### 2.1.1 Predict the physical and chemical properties of the candidate proteins

First, we used the Uniprot database (Sha et al., 2020) (https://www.uniprot.org/) to obtain the amino acid sequences of ESAT-6, PE35, PPE38 and PE4. Secondly, use ProtParam (http://web.expasy.org/protparam/) software to analyze the physical and chemical properties of the target protein sequence (Luo et al., 2025), including the number of amino acids, molecular formula, theoretical isoelectric point, instability index, and grand average of hydropathicity (GRAVY). If the instability index is less than 40, the protein is stable; if the GRAVY value is less than 0, the protein is hydrophilic.

#### 2.1.2 Homology prediction

The candidate proteins were compared in sequence with the human protein database (*Homo sapiens*, taxid:9606) using the NCBI BlastP database (https://blast.ncbi.nlm.nih.gov/Blast.cgi?PAGE=Proteins) to determine their homology to human proteins (Li et al., 2022).

#### 2.1.3 Antigenicity prediction

Utilize “VaxiJen v2.0” (https://www.ddg-pharmfac.net/vaxijen/) to conduct an antigenic analysis of candidate proteins and tMEV, with a threshold of 0.4 for selecting vaccine proteins (Li et al., 2025). If the software’s prediction result is greater than 0.4, the protein may be antigenic.

#### 2.1.4 Allergenicity prediction

The AllergenFP v.1.1 (https://ddg-pharmfac.net/AllergenFP/) was utilized to assess the allergenic potential of ESAT-6, PE35, PPE38 and PE4, and the designed vaccine (Fan et al., 2024).

#### 2.1.5 Toxicity prediction

ToxinPred2 (https://webs.iiitd.edu.in/raghava/toxinpred2/) was employed to assess the toxicity of candidate proteins (Yu et al., 2022).

#### 2.1.6 Signal peptide prediction

To ensure that the immune response elicited by the vaccine can accurately and efficiently target stable functional antigenic epitopes on pathogens or diseased cells, while avoiding non-protective or even harmful immune reactions induced against signal peptides, signal peptide sequences need to be excluded in subsequent analyses (Mo et al., 2023). The SignalP 5.0 server (Yu et al., 2021) (https://services.healthtech.dtu.dk/services/SignalP-5.0/) was utilized to predict the presence of signal peptides in candidate proteins, as well as the locations of their cleavage sites.

#### 2.1.7 Transmembrane helices prediction

Utilize DeepTMHMM - 1.0 (https://services.healthtech.dtu.dk/services/DeepTMHMM-1.0/) to determine the most probable positions and orientations of the transmembrane helices within the candidate protein (He et al., 2024).

#### 2.1.8 Screening of vaccine sequences

##### 2.1.8.1 T cell epitope prediction

Tuberculosis is prevalent worldwide, and China ranks third globally in terms of tuberculosis burden. As China’s westernmost region, Xinjiang has a relatively high tuberculosis incidence rate nationwide, ranking second across the country. The epidemic is especially severe in southern Xinjiang (Yilihamu et al., 2025). Therefore, this study conducts epitope prediction based on high-frequency alleles of populations in Xinjiang. Specifically, Cytotixic T cell epitopes (CTLEs) were predicted using the IEDB database (http://tools.immuneepitope.org/main/) and NetMHCpan-4.1 software (https://services.healthtech.dtu.dk/services/NetMHCpan-4.1/) with HLA-A*1101 (13.46%), HLA-A*0201 (12.50%) and HLA-A*0301 (10.10%); Helper T cell epitopes (HTLEs) were predicted using the IEDB database and NetMHCIIpan-4.0 software (https://services.healthtech.dtu.dk/services/NetMHCIIpan-4.0/) with HLA-DRB1*0701 (16.3%), HLA-DRB1*1501 (8.65%) and HLA-DRB1*0301 (7.69%) (Yin et al., 2023). Notably, the signal peptide sequence of the target protein was not considered during epitope screening. Subsequently, this study utilized the aforementioned tools—such as ProtParam, VaxiJen v2.0, AllergenFP v.1.1, and ToxinPred2—to analyze the physicochemical properties of these T-cell epitopes; based on these analyses, epitopes that were stable, hydrophilic, non-toxic, and non-allergenic were selected as optimal T-cell epitopes for constructing a recombinant multi-epitope vaccine.

##### 2.1.8.2 B cell epitope prediction

First, the ABCpred (https://webs.iiitd.edu.in/raghava/abcpred/) were used to predict B cell linear epitopes (LBEs) in ESAT-6, PE35, PPE38 and PE4, setting the epitope length to 16 mer. Secondly, the selected CBEs were predicted and visualized using the Ellipro tool from IEDB (https://www.iedb.org) (Shi et al., 2025). Subsequently, the same methods described in Section 2.1.8 were employed to screen for dominant B-cell epitopes for the construction of a recombinant multi-epitope vaccine.

#### 2.1.9 Vaccine connection

We selected antigenic, nontoxic, and hydrophilic dominant peptide epitopes and utilized different linkers to connect them in series. Specifically, the CTLEs are linked via the AAY linker, the HTLEs are connected through the GPGPG linker, the CTLEs and HTLEs are linked by the HEYGAEALERAG linker, and the CBEs are connected to the LBEs through the KK linker (Shi et al., 2024). To further enhance the immunogenicity of the peptide epitopes, we selected the adjuvant human beta-defensin-3 (UniProt database accession number: Q5U7J2), which possesses immunomodulatory effects, and linked it to the adjuvant antibody response PADRE sequence (Shang et al., 2024) via the EAAAK linker at the N-terminal. Additionally, a His-tag (HHHHHH) that was preceded by the RVRR linker was incorporated at the C-terminus to facilitate the purification of the expressed tMEV protein. Ultimately, the complete sequence of tMEV was obtained, and its hydrophilicity, allergenicity, stability, toxicity, and antigenicity were predicted.

#### 2.1.10 Vaccine solubility prediction

The solubility of tMEV vaccine was predicted using available E. coli Protein solubility data in the cell-free expression system protein-SOL (Chai et al., 2025) (https://protein-sol.manchester.ac.uk). If the predicted solubility is >0.58, it indicates that the protein has strong solubility in E.coli, facilitating subsequent selection of E.coli for transformation and induced expression of the vaccine protein.

#### 2.1.11 Immunostimulation

The C - ImmSim immune simulated (https://wwwold.iac.rm.cnr.it/~filippo/c-immsim/index.html) is used (Zhu et al., 2024). The random seed was set to 12345, with a simulation volume of 50 and simulation step length of 1050. The selected high frequency alleles were MHC-I-HLA-I *11:01, HLA-A*24:02, HLA-C*01:02, HLA-DQB1*03:01, HLA-DRB1*09:01, HLA-DRB1*15:01. The FASTA-formatted sequence was used as input for the system and administered in three doses at time units 1, 84, and 168 (each unit ≈8 hours), corresponding to real-world injections at week 0 (initial dose), week 4 (28-day interval), and week 8 (56-day interval).

#### 2.1.12 Prediction and optimization of the secondary and tertiary structures of MEV

The SOPMA online analysis software (http://npsa-pbil.ibcp.fr/cgibin/npsa_automat.pl?page=/NPSA/npsa_sopma.html) were used to predict and analyze the proportions and distributions of α-helices, beta bridges, beta angles, and random coils in proteins (Ma et al., 2025). This study employs AlphaFold2 (https://proteiniq.io/app/alphafold-2) to predict and model the tertiary structure of tMEV. The GalaxyWEB server (http://galaxy.seoklab.org/) was employed to optimize the tertiary structure of tMEV. The UCLA - DOE LAB - SAVES v6.0 tool (https://saves.mbi.ucla.edu) was utilized to verify the protein tertiary structure model and to integrate results from multiple servers, including ERRAT, Verify3D, and PROCHECK (Sadeghi et al., 2023).

#### 2.1.13 Molecular docking

The HDOCK server (Yan et al., 2020) (http://hdock.phys.hust.edu.cn/) was used to dock the tMEV vaccine peptide with TLR-4 to assess the affinity between tMEV and TLR-4. Select the model with the optimal binding affinity from the top ten ranked docking models by comparing docking scores. A more negative docking score indicates a higher binding probability. Molecules are highly likely to bind when the confidence score exceeds 0.7; molecules may bind when the confidence score ranges from 0.5 to 0.7; molecules are unlikely to bind when the confidence score is below 0.5. The docking results were visualized using PyMOL v2.5.0.

#### 2.1.14 Molecular dynamics simulation

Molecular dynamics simulations were conducted using GROMACS 2024.2 (Albutti, 2025). The specific procedure is as follows:the protein was modeled using the AMBER14SB-PARMBSC1.ff force field and the TIP3P water model. The simulation box boundaries were set to maintain a distance of 1.0 nm from the system atoms, with simulations conducted at 298.15 K, 1 atm pressure, and physiological salt concentration. The process began with energy minimization, using an emtol value of 100.0 kJ mol^-^¹ nm^-^¹; followed by a restricted dynamics simulation for the protein atoms, lasting 300 ps with a time step of 1 fs; finally, the production simulation was set to a duration of 100 ns with a time step of 2 fs. For Coulomb interactions during the simulation, the Particle Mesh Ewald (PME) method was employed, with a Coulomb cutoff radius of 1.0 nm. For van der Waals (vdW) interactions, a truncation method was applied, setting the vdW cutoff radius to 1.0 nm. The pressure coupling algorithm was specified as the Parrinello-Rahman method. Built-in GROMACS tools were utilized to calculate various parameters, including the ligand’s root-mean-square deviation (RMSD), Radius of Gyration (Rg), Solvent Accessible Surface Area (SAAS), H-bonds Number and Gibbs Energy Landscape.

### 2.2 Experimental methods

#### 2.2.1 Bacteria and culture method

*Escherichia coli DH5α* (Thermo Fisher Scientific, USA, Cat. No. 18265017) was cultured in Lysogeny Broth (LB) medium (BD Biosciences, USA, Cat. No. 244620) at 37 °C with agitation (200 r.p.m.). *Mycobacterium bovis BCG (ATCC 35734)* was maintained in Middlebrook 7H9 broth (BD Biosciences, Cat. No. 271310) supplemented with 0.05% Tween 80 (Sigma-Aldrich, USA, Cat. No. P1754) and 30 μg/mL kanamycin (Sigma-Aldrich, Cat. No. 60615), under static conditions at 37 °C.

#### 2.2.2 The construction of the recombinant plasmid pMV361-tMEV

The tMEV gene was amplified from the pET41a-tMEV plasmid and cloned into the pMV361 plasmid containing the hsp60 promoter using EcoRI and HindIII restriction sites. The purified 1416 bp fragment containing the target gene was successfully integrated into the vector. This plasmid construction was performed by China Youkang Company, and the synthetic recombinant plasmid has been obtained.

#### 2.2.3 Expression and validation of rBCG-tMEV

The recombinant plasmid pMV361-tMEV was electroporated into *Mycobacterium bovis* BCG (ATCC 35734) using an Eppendorf Electroporator 2510 (Eppendorf AG, Germany, Cat. No. 4309150003). Transformed BCG were subsequently cultured on Middlebrook 7H10 agar (BD Biosciences, USA, Cat. No. 283810) supplemented with 10% oleic acid-albumin-dextrose-catalase (OADC) enrichment (BD Biosciences, Cat. No. 211886), 0.5% Tween 80 (Sigma-Aldrich, USA, Cat. No. P1754), and 30 μg/ml kanamycin (Sigma-Aldrich, Cat. No. 60615) was used to select transformed strains carrying the antibiotic resistance gene for 2 weeks at 37 °C until A600 reaches 0.6-0.8.

Following selection, positive colonies were subcultured in Middlebrook 7H9 broth (BD Biosciences, Cat. No. 271310) containing identical supplements and incubated with agitation (180 r.p.m.) for an additional 2–3 weeks to achieve bacterial amplification. Transformants underwent thermal activation at 43 °C, followed by continuous induction at 37 °C for 7 days.The culture precipitates were then collected by centrifugation at 25 °C and 6000 r.p.m. for 15min.

The cultured cells were treated with PMSF (a serine protease inhibitor) and then ultrasonically lysed in an ice bath for 30 minutes using a Qsonica Q700 (USA) set at 40% amplitude, 2s on/3s off cycles. Lysates were clarified by ultracentrifugation (12000 r.p.m., 1 h, 4 °C; Beckman Coulter Optima XPN-80, USA). Supernatant proteins were resolved on 10% SDS-PAGE gels (Bio-Rad, USA, Cat. No. 4561033) and visualized by Coomassie Brilliant Blue R-250 staining (Thermo Fisher Scientific, Cat. No. 20278).

For His-tagged protein purification, supernatants were loaded onto a HisTrap HP column (Cytiva, USA, Cat. No. 17524801) pre-equilibrated with binding buffer (20 mM sodium phosphate, 500 mM NaCl, 20 mM imidazole, pH 7.4). After washing with 50 mM imidazole, recombinant tMEV was eluted using 150 mM imidazole (Sigma-Aldrich, Cat. No. I5513). Western blot verification utilized rabbit anti-His monoclonal antibody (1:2, 000; Abcam, UK, Cat. No. ab18184) as the primary antibody and HRP-conjugated goat anti-rabbit IgG (1:5, 000; Jackson ImmunoResearch, USA, Cat. No. 111-035-003) as the secondary antibody, with chemiluminescent detection (Pierce ECL Plus, Thermo Fisher Scientific, Cat. No. 32132).

#### 2.2.4 Immunization

Forty-eight female BALB/c mice (6-week-old, specific pathogen-free) were procured from the Experimental Animal Center of Xinjiang Medical University (China) and housed under controlled environmental conditions (22 ± 1 °C, 55% relative humidity, 12-hour light/dark cycle) with ad libitum access to autoclaved feed (Xietong Organism, China, Cat. No. SCXK2022-0003) and sterile water. All experimental protocols strictly adhered to the ARRIVE guidelines and were approved by the Institutional Animal Care and Use Committee (IACUC) of the First Affiliated Hospital of Xinjiang Medical University (Approval No.A240301-181), an AAALAC-accredited facility. Mice were randomly divided into four experimental groups (n = 12 per group. Each group was divided into two subgroups with six animals in each subgroup, for immune-index monitoring and challenge protection experiments respectively.): (1) The PBS control group received 50, 25, and 25 microliters of sterile phosphate-buffered saline (HyClone, USA, Cat.No. SH30256.01) at week 0, week 2 and week 4, respectively, (2) BCG group immunized with 1×10^6^ colony-forming units (CFU) of Mycobacterium bovis BCG Pasteur (ATCC 35734, USA) suspended in 100 μL PBS, (3) tMEV group administered 100 μg of a His-tagged multi-epitope vaccine (purified via HisTrap HP column, Cytiva, USA, Cat. No. 17524801) emulsified with 50% AddaVax™ adjuvant (InvivoGen, USA, Cat. No.vac-adx-10), and (4) rBCG-tMEV group receiving 1×10^6^ CFU of recombinant BCG expressing tMEV. The immunizing doses are shown in **Table 1**. Immunizations were delivered via subcutaneous multi-site injection using 29G insulin syringes (BD Biosciences, USA, Cat.No. 328438) at weeks 0, 2, and 4. Since durable pathogen-specific memory T-cell responses are a hallmark of effective vaccines, we evaluated short-term cellular immunity by aseptically isolating spleens four weeks post-final immunization and generating single-cell suspensions for intergroup comparison.

**Table 1:** The immunizing doses.

Group	Immunizing dose (0 week)	Immunizing dose (2 week)	Immunizing dose (4 week)
PBS	50μL	25μL	25μL
BCG	1 × 10^6^ cfu	1 × 10^3^ cfu	1 × 10^3^ cfu
tMEV	50μg	25μg	25μg
rBCG-tMEV	1 × 10^6^ cfu	1 × 10^3^ cfu	1 × 10^3^ cfu

#### 2.2.5 Challenge experiment

Four weeks following the final immunization (week 8 post-primary vaccination), mice were challenged with a low-dose aerosol containing 100 colony-forming units (CFU) of virulent *Mycobacterium tuberculosis* H37Rv (ATCC 27294, USA) using a Glas-Col inhalation exposure system (Glas-Col, LLC, Terre Haute, IN, USA; Model 099C A4212).

#### 2.2.6 ELISA

##### 2.2.6.1 ELISA experiment of mouse serum

Enzyme linked immunosorbent assay (ELISA) was used to determine specific IgG1 and IgG2a serum levels. At each designated time point, six mice per group were randomly selected for retro-orbital venous plexus puncture to collect serum samples, with measurements repeated three times to obtain the mean value. Briefly, 96-well polystyrene plates (Costar, USA, Cat. No. 3590) were coated with 100μL/well of the following antigens diluted in carbonate-bicarbonate buffer (pH 9.6, Sigma-Aldrich, USA, Cat. No. C3041): (1) PBS (negative control), (2) heat-killed Mycobacterium bovis BCG (1×10^5^ CFU/mL, ATCC 35734, USA), (3) recombinant tMEV protein (5 μg/mL, His-tagged; purity >95% by SDS-PAGE, Sino Biological, China, Cat. No. 11084-H08H), or (4) rBCG-tMEV lysate (1×10^5^ CFU/mL). After overnight incubation at 4 °C, plates were blocked with 3% (w/v) skim milk (BD Difco, USA, Cat. No. 232100) in PBS-T (0.05% Tween-20, Sigma-Aldrich, Cat. No. P7949) at 37 °C for 2 h.

Serum samples were initially diluted 1:50 in PBS-T and subjected to twofold serial dilutions. After 2 h incubation with diluted sera at 37 °C, plates were probed with horseradish peroxidase (HRP)-conjugated goat anti-mouse IgG (1:5, 000, Sigma-Aldrich, Cat. No. A0168), IgG1 (1:5, 000, Cat. No. A10551), or IgG2a (1:5, 000, Cat. No. A10685) for 1 h at 37 °C. HRP-conjugated activity was detected using TMB substrate (Thermo Fisher, USA; Cat. No. 34021) by incubating for 15 minutes in darkness. The reaction was terminated with 2 M H_2_SO_4_ (Sigma-Aldrich; Cat. No. 258105), and absorbance was measured at 450 nm (reference wavelength: 630 nm) using a SpectraMax M5 microplate reader (Molecular Devices, USA). Based on international standards for serological testing, the cutoff value is calculated as the mean optical density (OD) of negative-control samples plus twice the standard deviation (mean + 2 SD).

##### 2.2.6.2 ELISA experiment on human peripheral blood serum

The study enrolled 50 laboratory-confirmed active pulmonary tuberculosis patients (experimental group) and 50 healthy volunteers (control group). Tuberculosis diagnosis was established through sputum acid-fast staining, GeneXpert MTB/RIF, or mycobacterial culture positivity, with exclusion of individuals presenting immunodeficiency disorders, pregnancy, or other specific comorbidities. Healthy controls were required to exhibit normal chest X-ray findings, negative γ-interferon release assay (IGRA) results, and no history of infections or vaccinations within the preceding 3 months. Venous blood samples were collected from all participants, centrifuged to isolate serum, and stored at -80 °C for subsequent analysis. During ELISA procedures, purified tMEV antigen was coated at 10 μg/mL concentration, followed by 15-minute blocking with rapid blocking buffer. Serum samples diluted 1:100 were then added and incubated at 37 °C for 2 hours. HRP-conjugated anti-human IgG secondary antibody (Thermo Fisher Scientific; 31410; 1:5000 dilution) was subsequently applied for 1-hour incubation, with other experimental conditions maintained as previously described. Blank controls (antigen-free wells) and positive controls (serum with known high antibody titers) were systematically implemented throughout the experiment. Each sample was analyzed in three replicate wells, and final results were calculated as the mean optical density values.

#### 2.2.7 ELISPOT

Cellular immune responses were evaluated using enzyme-linked immunospot (ELISpot) assays to quantify IFN-γ, IL-2, TNF-α, granzyme B, perforin and IL-4 secretion. Polyvinylidene difluoride (PVDF) plates (MilliporeSigma, Burlington, MA, USA) were coated overnight at 4 °C with 5 μg/mL of capture antibodies specific for murine IFN-γ (clone XMG1.2; BD Pharmingen), IL-2 (clone JES6-5H4; BioLegend), TNF-α (clone MP6-XT22; eBioscience), granzyme B (clone GB11; R&D Systems), perforin (clone eBioOMAK-D; Invitrogen, Carlsbad) or IL-4(clone: 11B11; BD Pharmingen). After blocking with RPMI-1640 medium (Gibco, Carlsbad) containing 10% fetal bovine serum (HyClone, Logan), splenocytes (1×10^6^ cells/well) from immunized mice were stimulated with negative control, PBS, 1×10^6^ CFU/mL BCG, 10 μg/mL tMEV, 1×10^6^ CFU/mL rBCG-tMEV, or 10 μg/mL concanavalin A (ConA; Sigma-Aldrich; positive control) for 24 h at 37 °C under 5% CO_2_.

Following incubation, plates were washed with PBST (0.05% Tween-20) and incubated with biotinylated detection antibodies (BD Pharmingen) for 2 h at room temperature. Streptavidin-alkaline phosphatase (1:1000; Amersham Biosciences) was subsequently applied for 1 h. Spots were developed using BCIP/NBT substrate (Moss, Inc., Pasadena) for 40 min and quantified using an ELISPOT Bio Reader-4000 (BioSys GmbH, Karben). Data were expressed as spot-forming cells (SFC) per 10^6^ splenocytes after subtracting background values from negative controls.

#### 2.2.8 Flow cytometry

Spleens obtained from immunized mice were processed into single-cell suspensions using a 70 μm cell strainer (Corning, USA; Cat. No. 431751). Cells were labeled with fluorescent anti-mouse antibodies: Anti-Live/dead-BV421 (Thermo Fisher Scientific, USA; Cat. No. L34955), CD3-APC (BD Biosciences, USA; Cat. No. 561019), CD4-FITC (BD Biosciences; Cat. No. 553047), and CD8-PE-Cy5.5 (BD Biosciences; Cat. No. 551162). Cell counts were adjusted to 1×10^6^ cells/mL using Trypan Blue solution (Thermo Fisher, USA; Cat. No. 15250061), followed by analysis on a QuantObio flow cytometer (QuantObio, China; Model QF-2100). Data were analyzed with FlowJo v10 software (BD Biosciences; Cat. No. 633821). The specific gating strategy is as follows: first, gate lymphocytes based on forward scatter (FSC-A) and side scatter (SSC-A); second, exclude doublets (single-cell gate) using FSC-H and FSC-A; then select live cells according to live/dead staining; next, gate T cells within the CD3^+^ population; and finally identify CD4^+^ and CD8^+^ T cell subsets from the CD4 versus CD8 dot plot.

#### 2.2.9 Protection experiment

To enumerate bacteria in the in the lungs and spleens of mice after infection, the left lung (0.1 g) and part of the left spleen (0.1 g) from individual mice were homogenized with Gentle MACS M-tubes (Miltenyi Bio-tec) in 3 mL MilliQ water containing PANTA™ Antibiotic Mixture (BD Biosciences). The homogenate was serially diluted, plated and grown on 7H11 plates (BD Biosciences) for approximately 14 days at 37 °C and 5% CO_2_. CFU were counted, log-transformed to normalize data and shown as log10 CFU per whole organ tissue. In the experiment, 0.1 g of organ tissue was taken for grinding and CFU counting. Combined with the total weight of the intact organ, the total CFU value of the whole organ was obtained by conversion and extrapolation based on the colony count of 0.1 g organ tissue. Whenever possible a cutoff of 10 colonies were set to minimize variability and errors due to plating.

### 2.3 Statistical analysis

In this study, GraphPad Prism 10 software was used for statistical analysis of the data. After verifying the normality of the data via the Shapiro-Wilk test, the two-independent-samples t-test was used for comparison between the two groups. Data were expressed as mean ± standard deviation ($\bar{x} ± s$). One-way analysis of variance (ANOVA) combined with Tukey’s multiple-comparison test was adopted to evaluate differences among groups for multi-group comparisons, and data were presented as mean ± standard error. The significance level was set at α=0.05.

## 3 Results

### 3.1 Bioinformatics analysis

#### 3.1.1 Analysis of physicochemical properties, toxicity, allergenicity, and antigenicity, as well as human homology of candidate proteins

The antigenicity of the protein is represented by the VaxiJen score; a score greater than 0.4 indicates favorable protein antigenicity. Stability is assessed via the instability index, and a value below 40 denotes a stable protein. Hydrophilicity is characterized by the GRAVY value; a GRAVY value less than 0 means the protein is hydrophilic, with favorable solubility and accessibility to immune receptors. Proteins with good antigenicity, stability and hydrophilicity meet the criteria for screening candidate proteins for vaccine construction. Meanwhile, vaccine proteins constructed satisfying these conditions also facilitate subsequent expression and purification. Through comprehensive bioinformatic analysis of ESAT-6, PE35, PPE38, and PE4, we predicted favorable physicochemical characteristics (instability index<40, grand average of hydropathicity <0, non-toxic and non-sensitizing) and robust antigenicity (VaxiJen score >0.4) across all four proteins. Notably, the vaccine candidate proteins were compared against the human protein database (*Homo sapiens;* TaxID: 9606), and none of the four proteins exhibited sequence similarity to human counterparts, thereby minimizing potential autoimmune risks. As detailed in **Table 2**, the distinctive antigenic profiles and structural stability of these proteins informed their selection as core components in our rational vaccine design strategy targeting *Mtb*.

**Table 2:** Immunoinformatic characterization of *Mtb* vaccine candidates: the antigenicity, allergenicity, stability and homology.

Vaccine candidate	UniProt database accession number	System of T7SS	Antigenicity	Allergenicity	Stability	Human homology
ESAT-6	P9WNK7	ESX-1	0.6521	non-allergen	36.36	non-homology
PE35	P9WIG7	ESX-1	0.6201	non-allergen	18.25	non-homology
PE4	L7N661	ESX-3	0.4555	non-allergen	39.06	non-homology
PPE38	P9WHZ9	ESX-5	0.6202	non-allergen	33.99	non-homology

#### 3.1.2 Transmembrane helix and signal peptide analysis of the target protein

The SignalP 5.0 server was utilized to predict the presence of signal peptides in ESAT-6, PE35, PPE38, and PE4 proteins and the results indicated that none of them contained signal peptides (**Supplementary Figure S1A**). The DeepTMHMM-1.0 tool was employed to predict the outer membrane regions of ESAT-6, PE35, PPE38, and PE4 proteins. The findings revealed that all four proteins are located outside the membrane. In summary, the peptides from ESAT-6, PE35, PPE38, and PE4 are suitable candidates for vaccine design (**Supplementary Figure S1B**).

#### 3.1.3 Screening of vaccine sequences

##### 3.1.3.1 Prediction and verification of T cell epitope

Top 10 epitopes were selected based on MHC-I/II binding affinity (IC50 <50 nM) and subjected to biophysical profiling, retaining those demonstrating stability (instability index<40), hydrophilicity (GRAVY <0), strong antigenicity (VaxiJen >0.4), non-allergenicity, and non-toxicity(**Table 3**). Molecular docking using H-dock was performed to evaluate the binding affinity (ΔG ≤ -8.0 kcal/mol) of HTLEs with HLA-DR and the interaction stability (RMSD < 2.5 Å) of CTLEs with HLA-A/B supertypes, focusing on high-frequency HLA alleles in the Chinese population. Epitopes were linked using AAY/GPGPG spacers to optimize processing. Molecular docking revealed exceptional allele-specific binding: CTL-HLA-A24:02 complexes showed a docking score of -218.00 (confidence: 0.7958, ligand RMSD 115.23 Å), while HTL-HLA-DRB1*15:01 achieved -252.10 (confidence: 0.8851, RMSD 116.51 Å), confirming spatial complementarity critical for TCR recognition (**Supplementary Figure S2**). Population coverage analysis using IEDB tools predicted that T-cell epitope vaccines targeting tMEV achieved >98% global coverage across 17 key regions (including Asia, Africa, and others), based on high-frequency HLA alleles in China (class I/II allele frequency ≥5%). Notably, coverage rates in China for class I, class II, and combined epitopes all reached 99% or higher, confirming the vaccine’s broad-spectrum protective potential (**Supplementary Figure S3**).

**Table 3:** High confidence CTLs and HTLs epitopes after multi-parameter selection.

Epitope type	Protein	Residue numbers	Peptide sequence
CTLEs	ESAT-6	48-57	SEAYQGVQQK
		49-58	EAYQGVQQKW
		84-93	ASTEGNVTGM
	PE35	14-23	GTQVSDNALH
		65-74	NASAQDQLHR
	PE4	/	/
	PPE38	372-381	GANPHVVQAR
		378-387	VQARPSVVPR
HTLEs	ESAT-6	3-17	EQQWNFAGIEAAASA
		2-16	TEQQWNFAGIEAAAS
	PE35	/	/
	PE4	83-97	AGNAYAHAEAANAAR
		84-98	GNAYAHAEAANAARV
	PPE38	/	/

The epitope screening was conducted based on multi-criteria bioinformatics standards: (1) Structural stability (instability index threshold <40), (2) Hydrophilicity (GRAVY score <0 to exclude hydrophobic domains), (3) Immunological potency (VaxiJen antigenicity score >0.4 in bacterial antigen prediction model), with additional safety filters for (4) Non-allergenicity (AllergenFP v1.1) and (5) Non-toxicity (ToxinPred analysis). “/” indicates that there is no eligible peptide in the candidate epitope of the protein.

##### 3.1.3.2 Prediction of B cell epitope

All candidate epitopes were analyzed and the eligible epitopes are presented in **Table 4**. The three-dimensional structure of the selected LBEs epitope is shown in **Supplementary Figure S4**.

**Table 4:** High confidence LBEs and CBEs epitopes after multi-parameter selection.

Epitope type	Protein	Residue numbers	Peptide sequence
LBEs	ESAT-6	44-59	GGSGSEAYQGVQQKWD
	PE35	2-17	EKMSHDPIAADIGTQV
	PPE38	10-25	PEINSARIYAGAGSGP
		360-375	GAGMPAGMMGRGGANP
	PE4	456-471	PDSYPWVPSINPGLNF
		462-477	VPSINPGLNFYFGQPQ
CBEs	ESAT-6	75-84	NIRQAGVQYS
		41-52	AAWGGSGSEAYQ
		81-90	VQYSRADEEQ
		1-10	MAEMKTDAAT
		34-60	STAGSLQGQWRGAAGTAAQAAVVRFQE
		41-56	AAWGGSGSEAYQGVQQ
	PE35	/	/
	PPE38	/	/
	PE4	/	/

The epitope screening was conducted based on multi-criteria bioinformatics standards: (1) Structural stability (instability index threshold <40), (2) Hydrophilicity (GRAVY score <0 to exclude hydrophobic domains), (3) Immunological potency (VaxiJen antigenicity score >0.4 in bacterial antigen prediction model), with additional safety filters for (4) Non-allergenicity (AllergenFP v1.1) and (5) Non-toxicity (ToxinPred analysis). “/” indicates that there is no eligible peptide in the candidate epitope of the protein.

#### 3.1.4 Construction and physicochemical property analysis of tMEV vaccine

We selected antigenic, nontoxic, and hydrophilic dominant peptide epitopes and utilized different linkers to connect them in series. The complete sequence of tMEV was obtained, as illustrated in **Figure 1**. Following this, the hydrophilicity, allergenicity, stability, toxicity, and antigenicity of the tMEV vaccine protein were predicted. The results indicate that tMEV is a stable, hydrophilic, non-toxic, and non-allergenic protein, with its specific properties detailed in **Table 5**.

**Figure 1:**
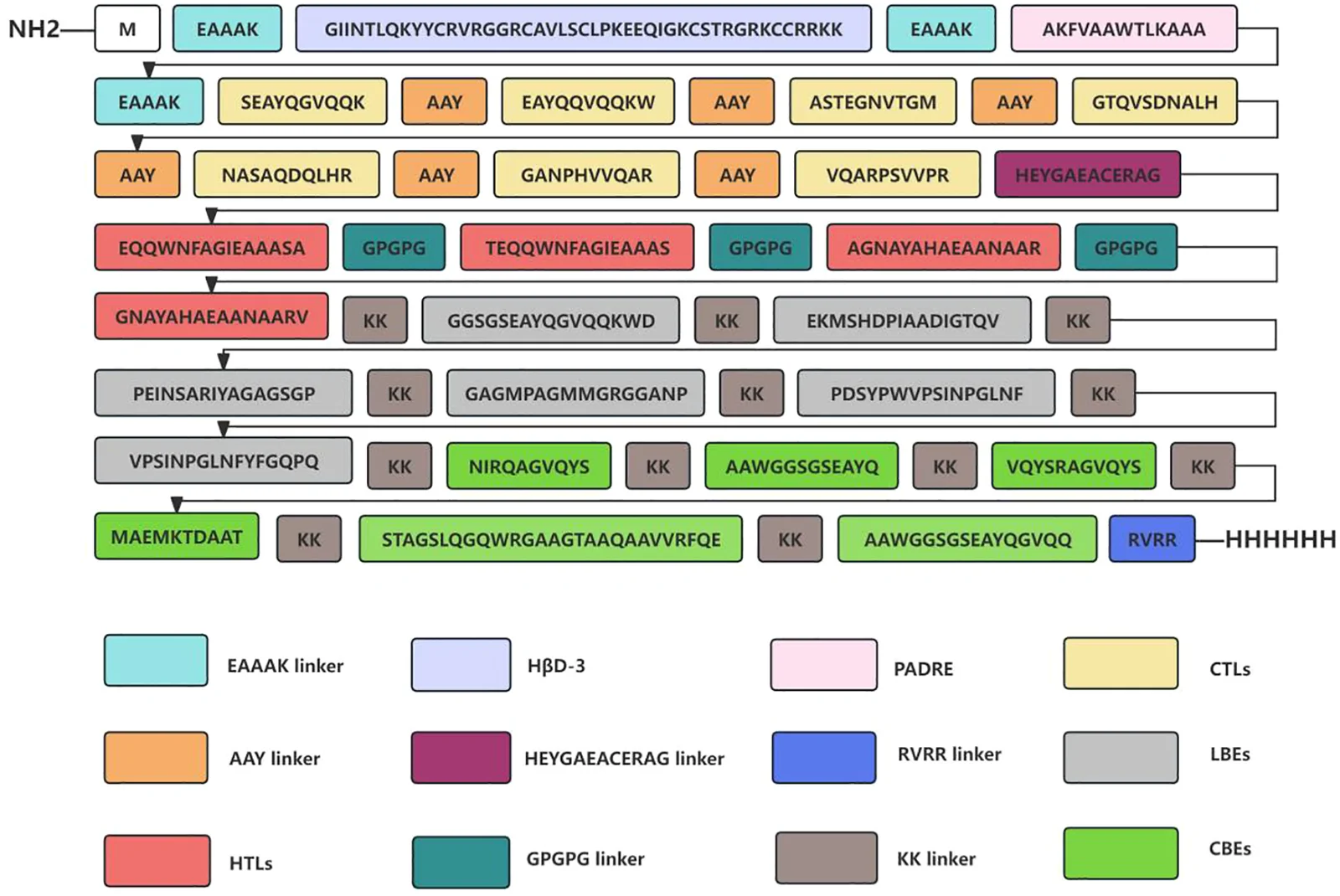
The design of the tMEV protein. The different colors in the image represent distinct tMEV structural components.

**Table 5:** Property analysis of tMEV.

Protein name	tMEV
Amino acid	464
Theoretical pI	9.77
Stability	Stable
Hydrophilicity	-0.699
Antigenicity	0.9442
Allergenicity	Non-allergen
Solubility	Good (**Supplementary Figure S5**)

#### 3.1.5 tMEV immune simulation

The C-ImmSim server was used to simulate the immune response following three injections of MEV. The total counts of macrophages (**Figure 2A**) and dendritic cells (**Figure 2B**) maintained tissue homeostasis at approximately 190–200 cells/mm³ throughout the simulation. RESTING cells constituted the major population, while ACTIVE, INTERNALIZED, and PRESENTING-1/2 states rose transiently upon antigen stimulation only during the early priming phase (0–50 days) and then rapidly declined. This indicates that MA/DCs immediately uptake, process and present antigens after antigen exposure, and return to a resting surveillance state once immune responses are initiated. The total count of helper T cells peaked at approximately day 30 during clonal expansion (around 12, 000 cells/mm³) (**Figure 2C**), with synchronized peaks of ACTIVE and DUPLICATING states (**Figure 2D**), reflecting robust proliferation after T-cell receptor activation. The effector population then contracted rapidly and stabilized at a memory/resting pool of roughly 1, 000 cells/mm³ after day 120. Memory TH cells (TH Mem) declined slowly after the peak and persisted at about 1, 200 cells, whereas the ANERGIC state was nearly absent, suggesting the immune response was dominated by effective activation without substantial tolerance induction. **Figure 2E** (total B cells and antibody isotypes) and **Figure 2F** (functional states of B cells) demonstrate that B cells peaked at approximately day 30 (around 780 cells/mm³), with concurrent increases in ACTIVE and DUPLICATING states accompanied by transient antigen internalization and presentation. This shows B cells are activated and proliferate as effector cells while also functioning as APCs for T-cell help. Total B cells subsequently persisted at roughly 400 cells/mm³, memory B cells (B Mem) accumulated gradually to around 100 cells, and B-cell isotypes underwent class switching from early IgM predominance toward IgG1/IgG2, indicative of germinal center affinity maturation and class-switch recombination. **Figure 2G** shows antigen (Ag) was rapidly cleared after three exposure peaks. IgM antibodies peaked first and decayed quickly, whereas IgG1/IgG2 peaked at approximately 40–50 days, then declined slowly and persisted long-term, verifying the transition of humoral immunity from an early IgM response to sustained protective IgG immunity. **Figure 2H** illustrates that IFN-γ peaked after three antigen challenges, accompanied by synchronous elevations of proinflammatory cytokines including TNF-α, IL-6 and IL-1β. IL-2 reached its peak following the third antigen stimulation, while IL-4 remained at relatively low levels, indicating the overall response was dominated by Th1-type cytokines alongside acute-phase inflammation. The involvement of regulatory factors such as TGF-β and IL-10 facilitated later resolution of inflammation and restoration of immune homeostasis. Collectively, this immune simulation predicts that the recombinant vaccine may enhance anti-tuberculosis immunity by coordinating macrophage activation, T/B lymphocyte responses, and the antibody-cytokine network. However, it is worth noting that The C-ImmSim server is a computational model for simulation and predictive analysis only. The actual immune status still needs to be analyzed and verified through *in vivo* and *in vitro* experiments such as ELISA and flow cytometry.

**Figure 2:**
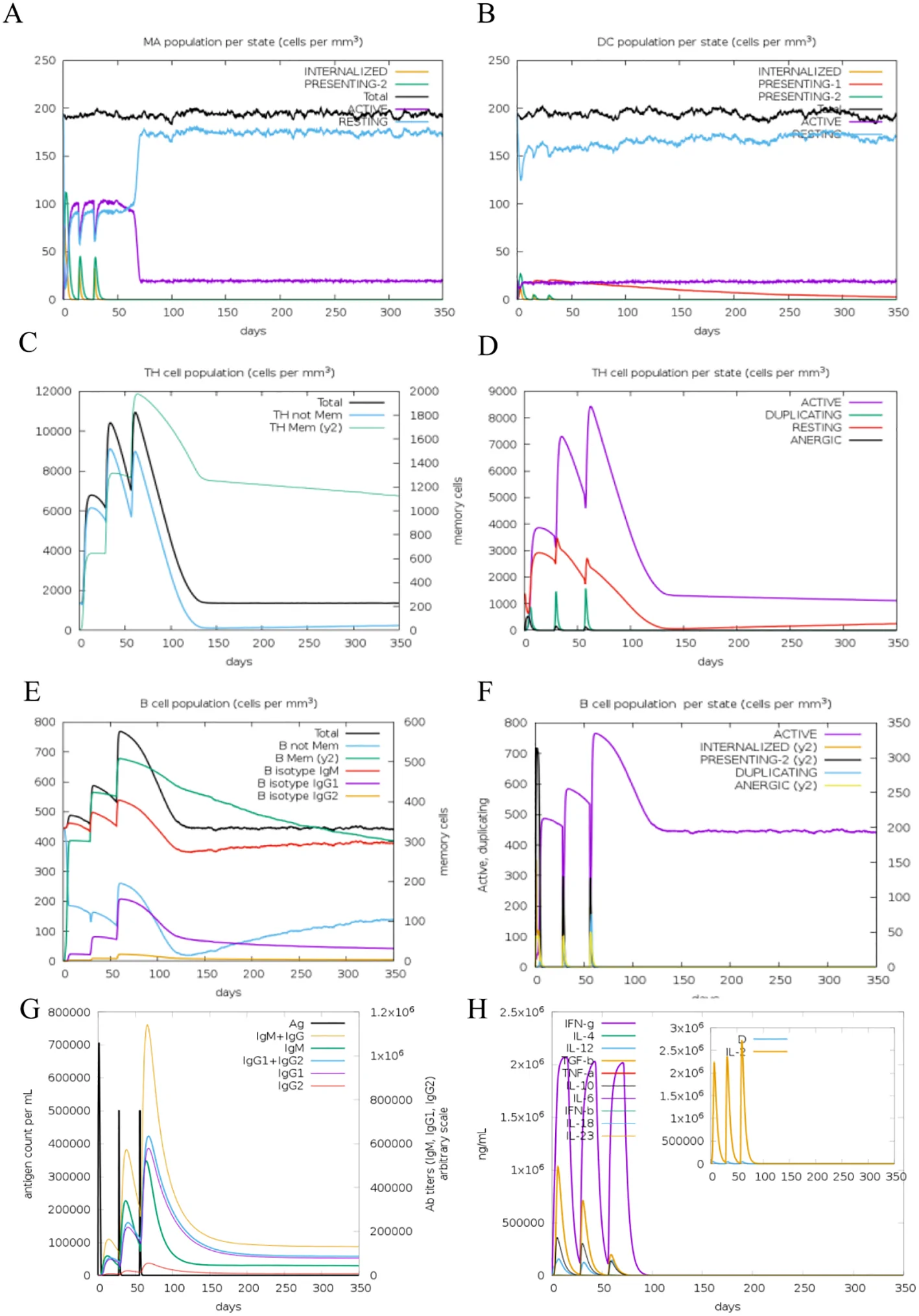
tMEV immune simulation. **(A)** Macrophage total count; internalized, presenting on MHC class II; active and resting macrophages; **(B)** Dendritic cells. DCs can present antigenic peptides on both MHC class-I and class-II molecules. The curves show the total number of bacteria broken down into active, resting, and internalized states and the corresponding. **(C)** The total number of TH cells; **(D)** TH cell population per state; **(E)** The total number of B cells; **(F)** B cell population per state. **(G)** Antibodies are subdivided according to isotype; **(H)** Concentrations of cytokines and interleukins. D in the inset plot is the danger signal.

#### 3.1.6 Prediction, refinement and validation of secondary and tertiary structures

The structural integrity of tMEV was systematically validated through a multi-tiered computational pipeline: Secondary structure predictions using SOPMA and PSIPRED 4.0 revealed a composition of 50.86% α-helices, 6.47% β-turns, 36.85% random coils, and 5.82% extended strands, with the high helical content (50.86%) and flexible regions (36.85% coils) suggesting both structural stability and functional accessibility (**Supplementary Figure S6**).

Tertiary structure modeling was achieved through AlphaFold2 prediction followed by GalaxyWEB refinement and SAVES v6.0 validation, generating a high-confidence 3D model (**Figure 3A**). After four refinements of the tertiary structure of tMEV, the final model achieved a GDT-HA parameter of 0.976, an RMSD of 0.324, a MolProbity score of 1.134, a clash score of 3.4, 0.0 poor rotamers, and 98.3% of residues in the favored region of the Ramachandran plot. Some faulty amino acid sites in the refined model were reconstructed through two local site reconstructions, resulting in a Ramachandran plot from PROCHECK confirming that 98.4% of residues were in the most favorable region and 1.6% in the additional allowable region, with a structure quality score of 97.4716 (**Figure 3B**), indicating that the tertiary structure is expected to have good model quality. The ProSA-web Z score was -7.42, indicating good overall model quality. Crucially, hierarchical consistency was observed between structural tiers–the tertiary structure retained 48.91% α-helical content (vs 50.86% predicted), while 89% of β-strand positions aligned with secondary structure predictions, and disordered coil regions corresponded to surface-exposed linker domains critical for epitope presentation.

**Figure 3:**
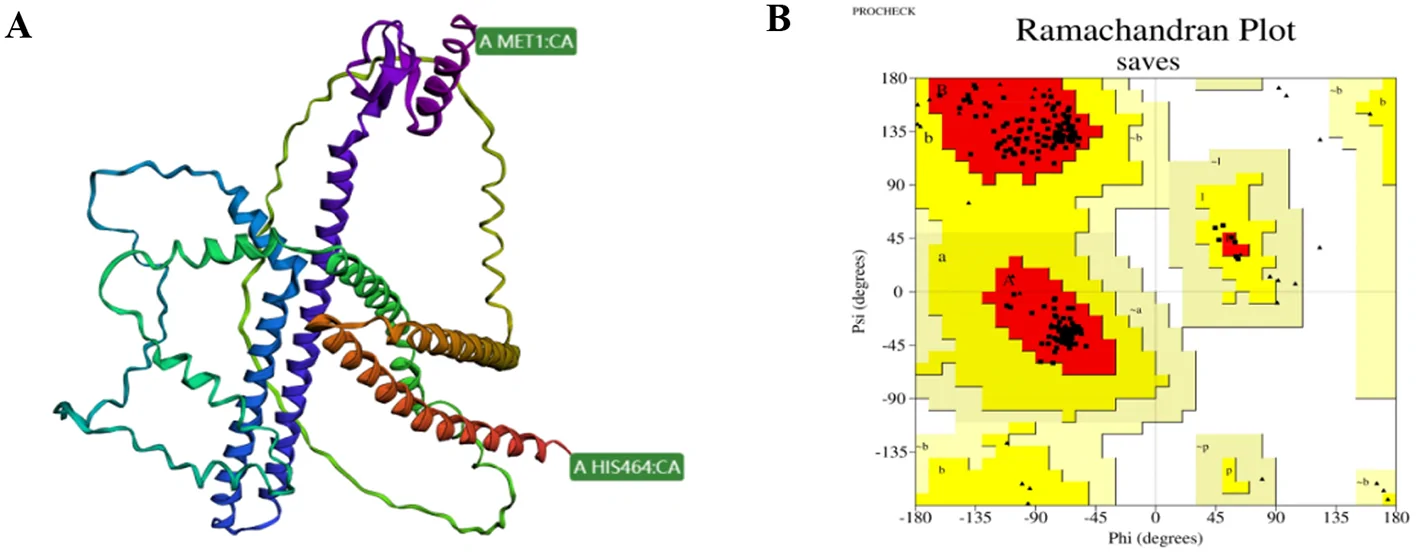
tMEV 3D model. **(A)** Diagram from the Robetta software tertiary structure. In the image, red represents CBEs, blue represents LBEs, green represents CTLs, and purple represents HTLs; **(B)** Ramachandran plot. The more amino acids in the red region, the more credible the tMEV 3D model is.

#### 3.1.7 The interaction and molecular dynamics simulation of tMEV and TLR-4

To predict the interaction characteristics of tMEV with TLR4, multi-scale computational biology approaches were employed: the complex model was constructed using AlphaFold v2.3.2, and the docking conformation was visualized via PyMOL v2.5.0 (**Figure 4**). A total of 132 action sites have been identified by Arpeggio analysis, which include 51 hydrogen bonds, 45 weak hydrogen bonds, 18 van der Waals forces, 23 charge interactions, 2 aromatic ring atomic interactions, and 49 hydrophobic interactions. It is noteworthy that multiple interactions occur at some of these sites.

**Figure 4:**
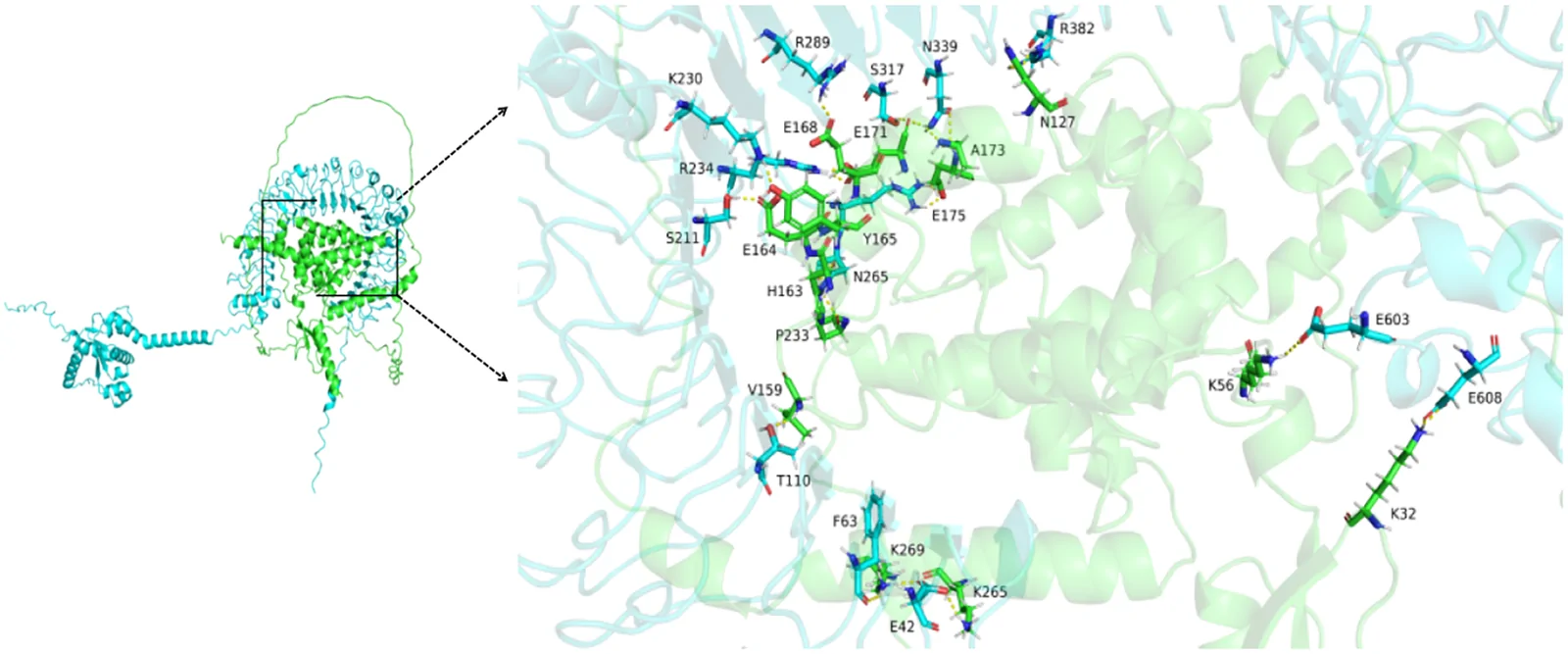
Computational characterization of TLR-4 / tMEV vaccine interaction. Blue: Structural domains of TLR-4 receptor (PDB ID 3FXI). Green: Engineered epitope clusters of tMEV vaccine. Yellow: Hydrogen bond force. Methodology: The complex was predicted using AlphaFold v2.3.2 with default multimer settings. Molecular docking conformations (lowest energy state shown) were visualized in PyMOL v2.5.0, with hydrogen bonds and electrostatic surfaces rendered resolution.

We performed a 100 ns continuous molecular dynamics simulation of the docked complex of tMEV and TLR-4 using the GROMACS 2024.2 tool. 100 ns is sufficient to observe local conformational changes and the stability of binding interfaces, yet it may fail to capture large-scale domain rearrangements, which require simulations of longer duration. Meanwhile, we performed three independent MD replicates with distinct initial velocity distributions, and consistent trends were observed across all replicates. RMSD reflects the conformational fluctuation of proteins and can be used to evaluate the binding stability between ligands and target proteins: a smaller value indicates more stable binding. In this study, during 0–20 ns: the three RMSD curves rose rapidly in tandem, which represented significant conformational relaxation of the initial system structure, and the protein, ligand and complex all deviated sharply from the initial crystal conformation; during 20–100 ns: all curves reached a plateau with minor fluctuations and stable values, where the RMSD of the complex remained steady at 2.8–3.0 nm, that of the protein ranged from 2.6–2.8 nm, and the ligand exhibited a slightly lower RMSD value. These computational prediction results demonstrate that the system achieved dynamic equilibrium after 20 ns, and the simulation data obtained in the subsequent 80 ns can be adopted for conformational statistical analysis, verifying that the overall backbone structure of the protein-ligand complex is stable (**Figure 5A**). Rg reflects the overall compactness of proteins; a higher value indicates a more extended and loose protein structure. From 0 to 20 ns, the Rg value drops rapidly from the initial 5.4 nm to 3.8–4.0 nm, which means the protein undergoes obvious folding and structural contraction at the early stage of simulation, transforming from an extended conformation to a compact one. During 20–100 ns, Rg fluctuates within a narrow range and stabilizes at 3.75–4.0 nm with no sustained upward or downward trends. This demonstrates that the tertiary structure of the protein maintains stable compactness in the equilibrium phase, without unfolding or structural expansion, and the overall folded conformation remains intact (**Figure 5B**). SASA describes the interactions between protein molecules and surrounding water molecules, revealing variations in protein hydrophilicity and hydrophobicity as well as reflecting the structural stability of proteins. From 0 to 60 ns, the SASA value decreases continuously and markedly, gradually dropping from an initial value of approximately 850 Å² to roughly 600–620 Å². Combined with the variation trend of Rg, this indicates that hydrophobic residues of the protein rapidly fold inward at the early stage of simulation to reduce the contact area with the solvent, with hydrophobic interactions driving protein folding. During 60–100 ns, the SASA value fluctuates slightly and plateaus, which demonstrates that the hydrophobic core of the protein becomes stably buried and the hydrophilic surface is fixed after equilibration. No sustained changes occur at the protein-solvent contact interface, and large-scale conformational rearrangements of the protein cease (**Figure 5C**). Hydrogen bonds are among the strongest non-covalent interactions. The number of hydrogen bonds formed during simulation reflects the binding strength. The number of hydrogen bonds rises rapidly from 0 to 40 ns, increasing from fewer than 10 at the initial stage to more than 50, which indicates that the ligand gradually forms stable hydrogen bond interactions with amino acids in the protein binding pocket at the early stage of simulation. From 40 to 100 ns, the number of hydrogen bonds remains at a high level (40–60) with continuous fluctuations, and abundant hydrogen bonds are sustained all the time, demonstrating that the ligand binds to the protein to form numerous stable intermolecular hydrogen bonds and maintain the stability of the complex (**Figure 5D**). Additionally, the MMGBSA values for protein-protein interactions remained stable throughout the simulation, maintaining an approximate value of -45 kJ/mol. The narrow blue channel with the lowest free energy is distributed along the direction of increasing PC1 and decreasing PC2, featuring only a single continuous low-energy conformational basin without multiple independent low-energy wells. This is consistent with the three-dimensional conformational dynamics, and there are no multiple conformational isomers with similar energies. Conformational changes transition gently along PC1/PC2 without high-energy barriers hindering conformational transitions. This prediction aligns with the equilibrium results of RMSD, Rg and SASA. After system equilibration, the system converges to a single dominant conformation with low conformational heterogeneity, indicating uniform and stable conformations of the complex (**Figures 5E, F**).

**Figure 5:**
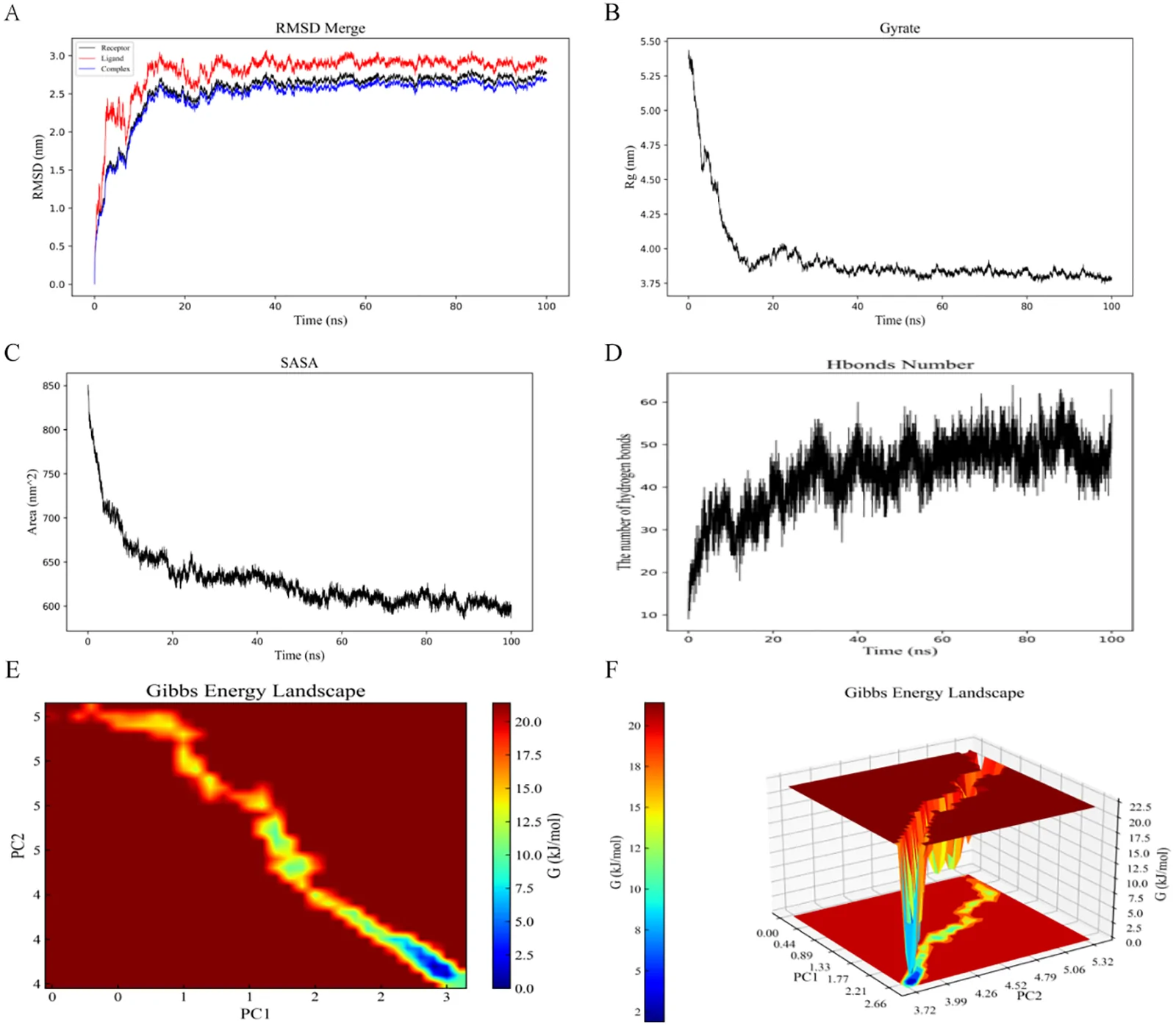
Molecular dynamics simulation. **(A)** RMSD trajectories. **(B)** Radius of gyration (Rg) reduction. **(C)** Solvent-accessible surface area (SASA = 600 nm²). **(D)** Hydrogen bond persistence at the binding interface. **(E)** 2D. Free energy basin distribution for tMEV (concentrated) versus unmodified system (dispersed). **(F)** 3D. Free energy basin distribution for tMEV (concentrated) versus unmodified system (dispersed).

### 3.2 Experimental results

Based on the design and predictions regarding tMEV, it appears to be a promising vaccine protein that is stable and capable of stimulating both humoral immune responses and cellular immunity. To verify the consistency of tMEV with these predicted outcomes, we conducted both *in vivo* and *in vitro* experiments. Furthermore, we expressed tMEV in BCG to assess whether the rBCG-tMEV vaccine offers improved efficacy compared to BCG alone.

#### 3.2.1 Expression and identification of tMEV

To confirm successful tMEV expression in rBCG-tMEV, Transformants underwent thermal activation, His-tagged tMEV was purified from ultrasonically disrupted cells using nickel-affinity chromatography, achieving optimal purity and yield at 150 mM imidazole elution. The expected molecular weight of the protein is approximately 51 kDa. SDS-PAGE analysis and Western blot analysis using an anti-His tag antibody confirmed the successful expression of tMEV in recombinant BCG (**Figure 6**).

**Figure 6:**
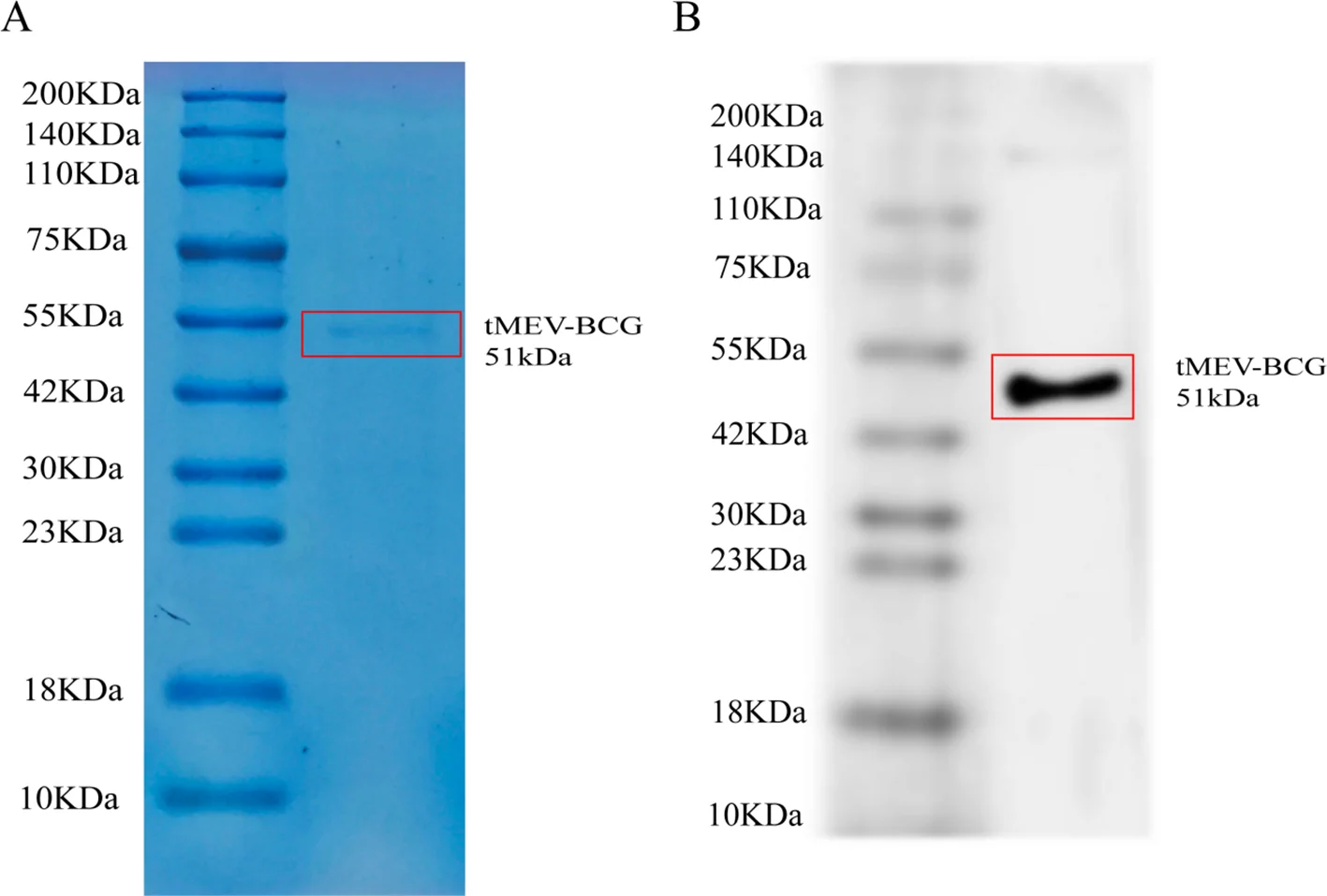
Expression analysis of recombinant tMEV in BCG. **(A)** Coomassie Brilliant Blue-stained SDS-PAGE. Lane 1: Protein molecular weight marker. Lane 2: His-tagged tMEV (~51 kDa) eluted with 150 mM imidazole. **(B)** Western blot. Lane 1: Protein molecular weight marker. Lane 2: His-tagged tMEV (~51 kDa) using anti-His antibody.

#### 3.2.2 ELISA

##### 3.2.2.1 ELISA experiment on human peripheral blood serum

To validate the specificity of the tMEV vaccine, this study employed an enzyme-linked immunosorbent assay (ELISA) to compare antibody responses to tMEV between serum samples from *Mycobacterium tuberculosis*-infected individuals (n=50) and healthy controls (n=50). The results indicated that tMEV exhibited significantly higher IgG-binding activity in tuberculosis patients (OD450 = 0.780 ± 0.551) compared to the healthy group (OD450 = 0.466 ± 0.118; *P* < 0.001, two-tailed t-test) (**Figure 7**). These findings suggest that tMEV is recognized by tuberculosis-associated antibodies. It is worth noting that human ELISA test results reflect antigen-recognition capacity rather than diagnostic performance.

**Figure 7:**
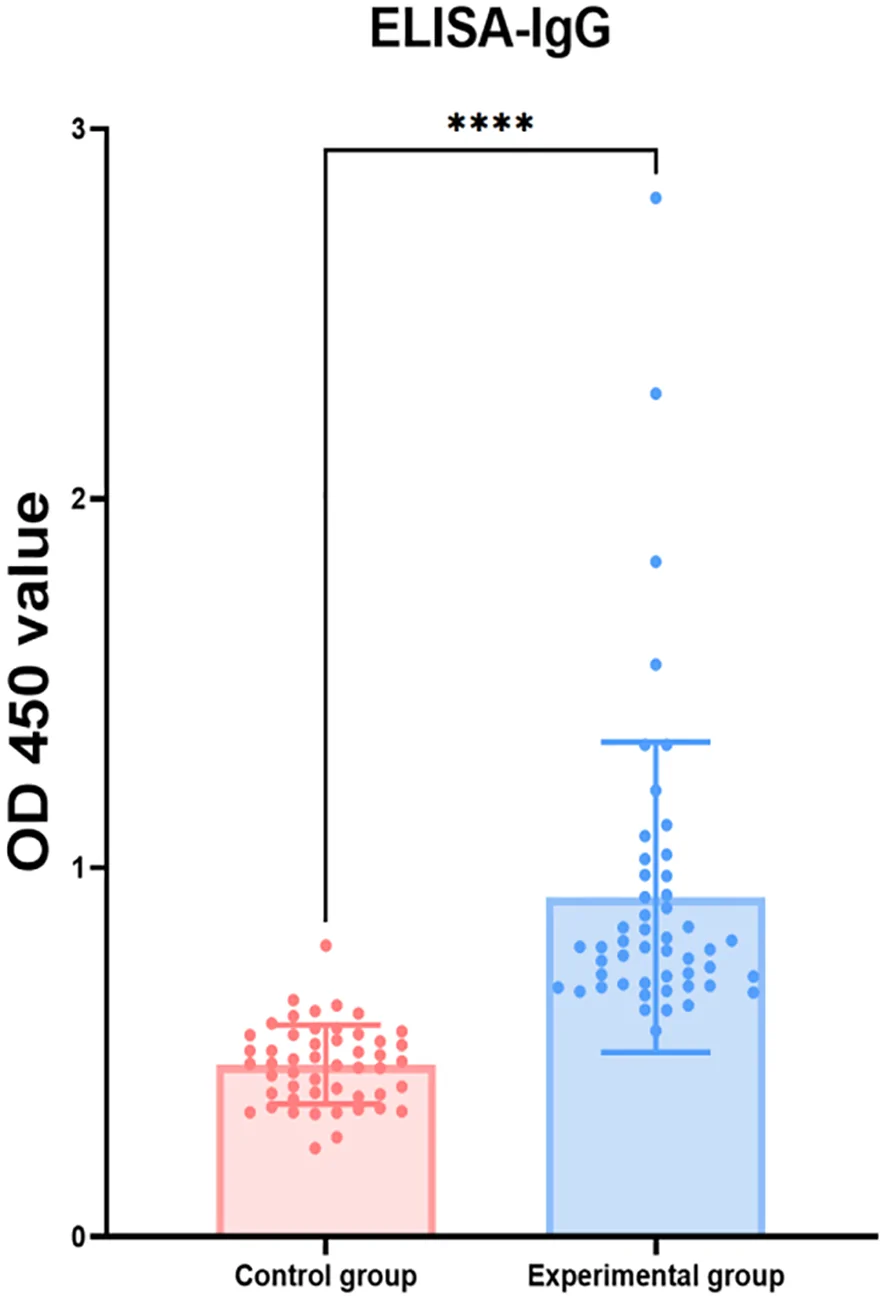
Comparison of IgG-binding activity to tMEV between tuberculosis patients (Experimental group) and healthy controls (Control group). Data are presented as a bar graph illustrating mean OD450 values ± standard deviation (SD), with a significant difference (****P < 0.0001) determined by two-tailed unpaired t-test between the tuberculosis patient group (n = 50) and healthy control group (n = 50).

##### 3.2.2.2 ELISA experiment of mouse serum

To assess the vaccine’s capacity to elicit humoral immunity in mice, serum IgG1 and IgG2a antibody titers were quantified via ELISA. The optical density (OD450) values are expressed as the mean logarithmic value of antibody titers. Compared to the PBS control group, all vaccine groups (tMEV, BCG, and rBCG-tMEV) exhibited significantly elevated IgG1 (**Figure 8B**, *P* < 0.05) and IgG2a (**Figure 8C**, *P* < 0.05) antibody levels, indicating robust systemic humoral immune activation. While rBCG-tMEV showed no statistically significant difference in IgG1/IgG2a induction compared to BCG alone, a trend toward higher IgG1 production was observed in the rBCG-tMEV group.

**Figure 8:**
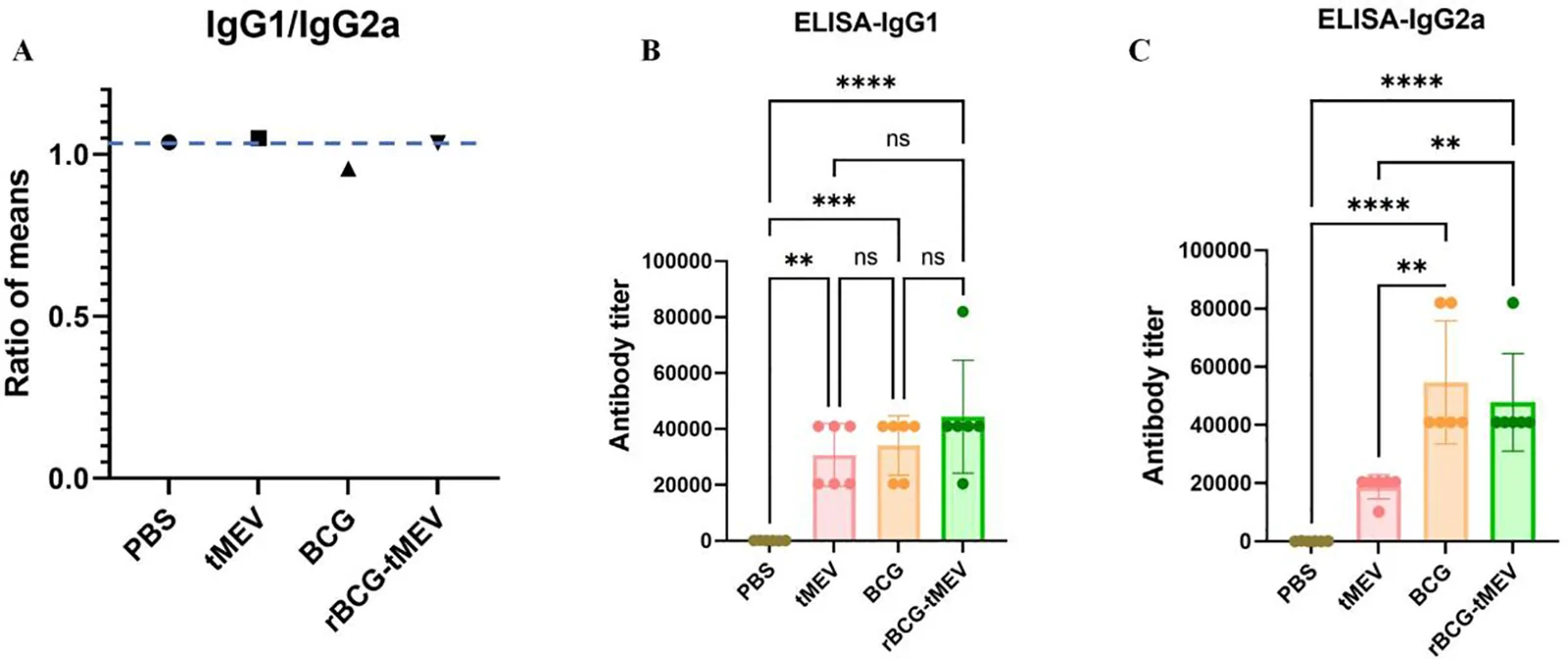
Analysis of serum IgG1 and IgG2a antibody profiles in immunized mice. **(A)** IgG1/IgG2a ratio across experimental groups, with the blue dashed line indicating the baseline ratio in the PBS control group. **(B)** Log10-transformed IgG1 antibody titers. **(C)** Log10-transformed IgG2a antibody titers. The data are expressed as mean ± SEM (n=6 per group). Following the verification of data normality using the Shapiro-Wilk test, a univariate analysis of variance (ANOVA), along with the Tukey multiple comparison method, was employed to assess the differences among the groups. The statistical analysis was conducted using GraphPad Prism. ns: not significant, *P* ≧0.05; **P <0.01; ***P <0.001; ****P <0.0001.

In mice, Th1 cells drive IgG2a antibody production, while Th2 cells primarily generate IgG1 antibodies. Compared to the PBS control group, tMEV induced a Th2-biased immune response, BCG triggered a Th1-skewed response, and rBCG-tMEV promoted a mixed Th1/Th2 profile. Importantly, IgG2a antibody induction correlated positively with vaccine efficacy. Both tMEV and rBCG-tMEV significantly elevated IgG2a levels compared to the PBS group (P < 0.001), suggesting their capacity to elicit effective humoral immune responses in mice. (**Figure 8A**).

#### 3.2.3 Flow cytometry

Following confirmation of tMEV expression, we evaluated the immunogenicity of rBCG-tMEV by immunizing mice with PBS, tMEV, BCG, or rBCG-tMEV. Flow cytometric analysis of splenocytes four weeks post-immunization revealed distinct T-cell activation profiles. Compared to PBS, tMEV, BCG, and rBCG-tMEV significantly elevated CD4^+^T-cell frequencies (*P* < 0.05), with rBCG-tMEV outperforming parental BCG (*P* < 0.05). While BCG and rBCG-tMEV also induced CD8^+^T-cell expansion versus PBS (*P* < 0.05), tMEV alone failed to significantly augment CD8^+^ levels. Notably, rBCG-tMEV showed a non-significant trend toward higher CD8^+^ T cells than BCG (*P* = 0.056). Overall, tMEV suggested a stronger capacity for CD4^+^ T cell activation, BCG showed greater effectiveness in activating CD8^+^ T cells, and rBCG-tMEV may integrate the advantages of both (**Figure 9**).

**Figure 9:**
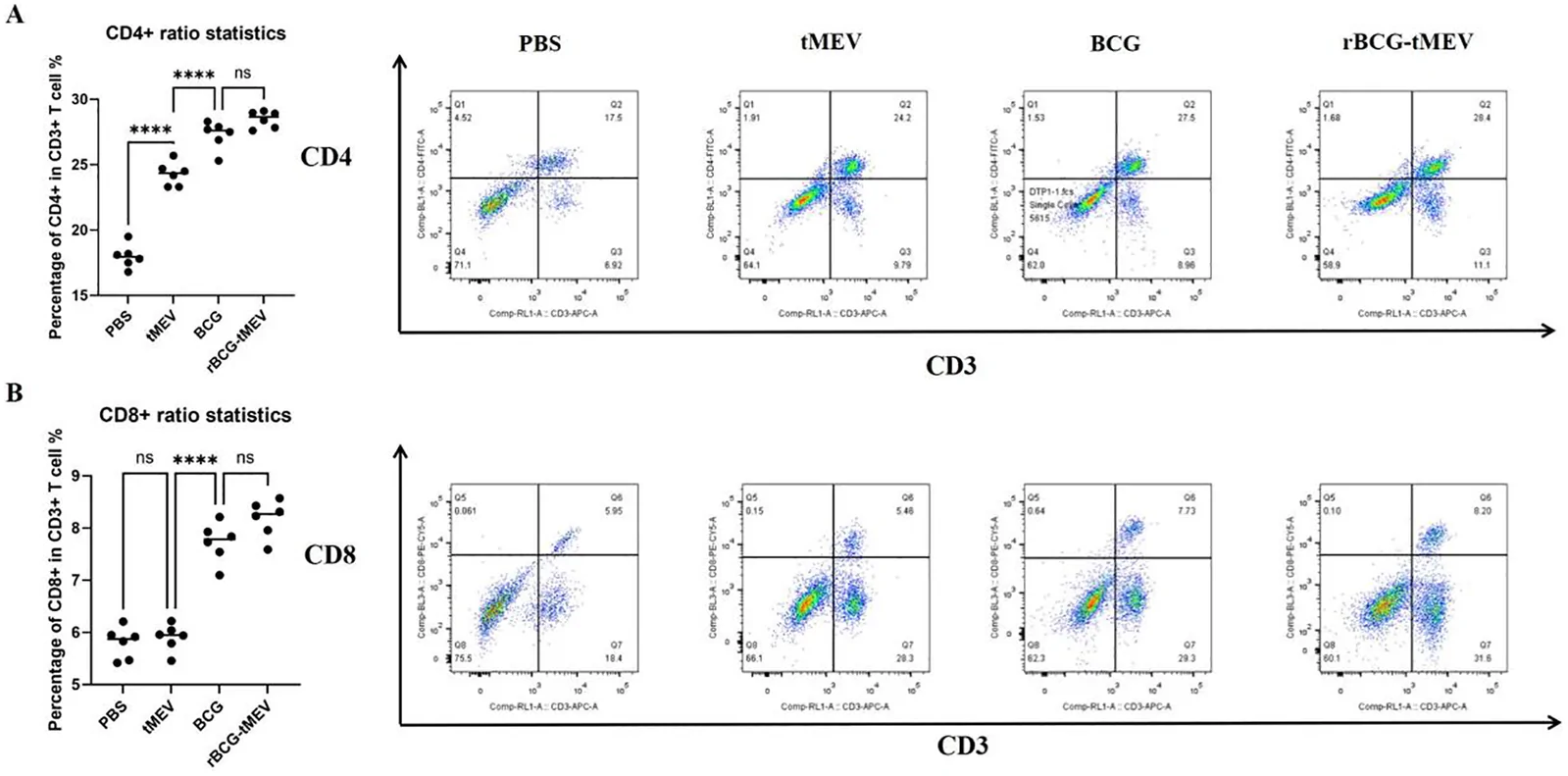
Analysis of T-cell activation in splenocytes from PBS-, tMEV-, BCG-, and rBCG-tMEV-immunized mice. **(A)** Left: Quantification of CD4^+^ T cells as a percentage of CD3^+^ T cells; Right: Representative flow cytometry gating strategy for CD4^+^ T cells (CD3^+^CD4^+^). **(B)** Left: Quantification of CD8^+^ T cells as a percentage of CD3^+^ T cells; Right: Representative flow cytometry gating strategy for CD8^+^ T cells (CD3^+^CD8^+^). The data are expressed as mean ± SEM (n=6 per group). Following the verification of data normality using the Shapiro-Wilk test, a univariate analysis of variance (ANOVA), along with the Tukey multiple comparison method, was employed to assess the differences among the groups. The statistical analysis was conducted using GraphPad Prism. ns: not significant, *P*≧0.05; ****P <0.0001.

#### 3.2.4 ELISPOT

Four weeks post-immunization, splenic lymphocytes were isolated from mice and stimulated with phosphate-buffered saline (PBS), tMEV, BCG, or recombinant rBCG-tMEV. ELISPOT assays revealed that tMEV stimulation significantly enhanced Th1-type immune responses compared to PBS, with increased spot-forming cell (SFC) counts for IFN-γ (**Figure 10A**), IL-2 (**Figure 10B**), and TNF-α (**Figure 10C**) (all *P* < 0.0001), indicating tMEV’s potential to activate baseline protective immunity. However, conventional BCG vaccination indicated superior Th1-inducing capacity, elevating IFN-γ, IL-2, and TNF-α secretion levels by 65.39%, 50.21%, and 74.66% (all *P* < 0.001), respectively, compared to tMEV, suggesting insufficient immunoprotection from tMEV alone.

**Figure 10:**
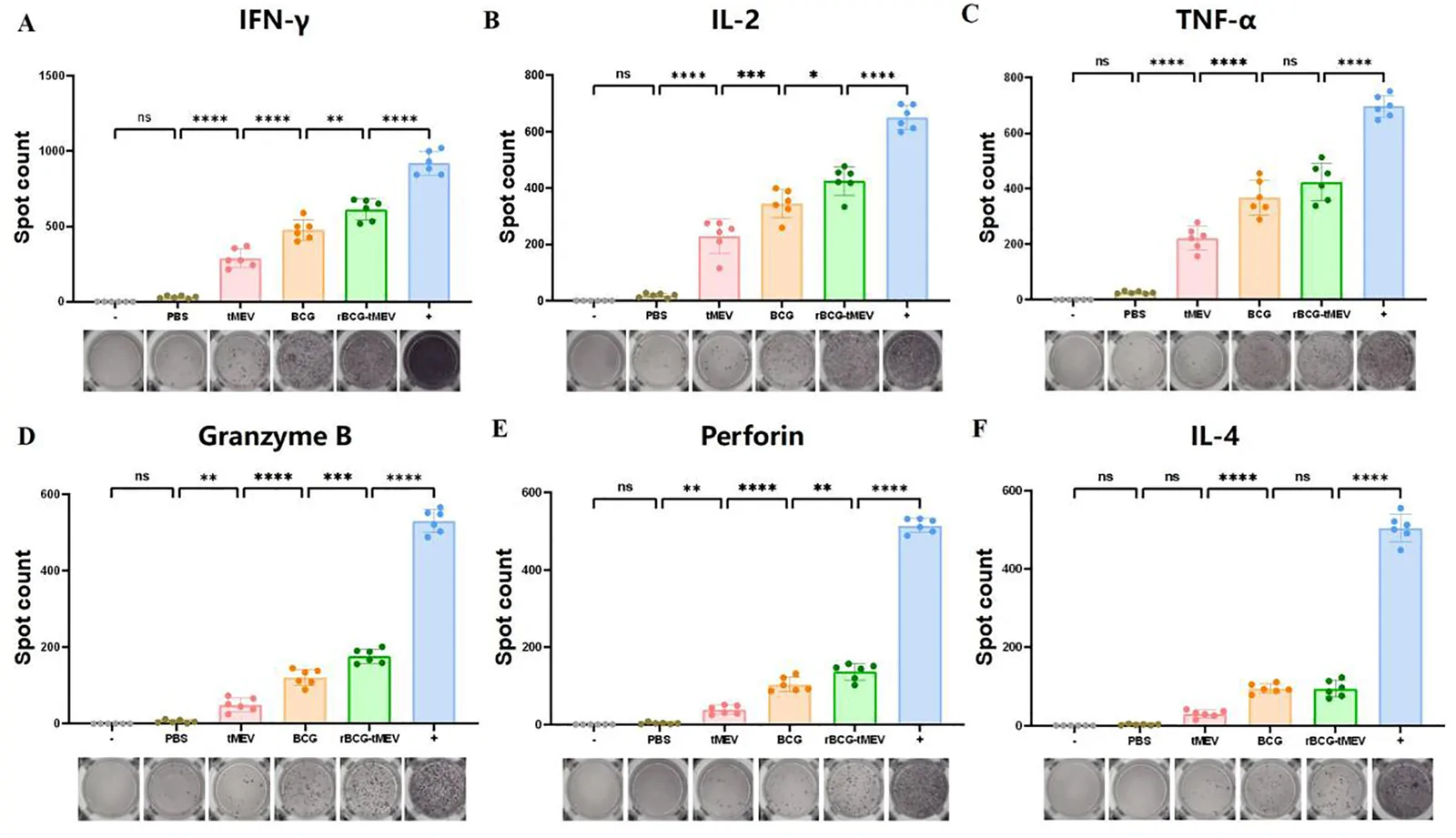
ELISPOT assay for antigen-specific immune responses: Spleen cells were harvested at week 4 post-last immunization and stimulated for detection. **(A)** Representative ELISPOT images of IFN-γ (down) and quantitative spot-forming cell (SFC) counts (up). **(B)** Representative ELISPOT images of IL-2 (down) and quantitative SFC counts (up). **(C)** Representative ELISPOT images of TNF-α (down) and quantitative SFC counts (up). **(D)** Representative ELISPOT images of Granzyme B (down) and quantitative SFC counts (up). **(E)** Representative ELISPOT images of Perforin (down) and quantitative SFC counts (up). **(F)** Representative ELISPOT images of IL-4 (down) and quantitative SFC counts (up). Experimental groups: − denotes the negative control group (PBS, tMEV, BCG, rBCG-tMEV); + denotes the positive control group. Data are presented as mean ± standard error of mean (SEM) (n = 6 per group). After verifying the normality of the data using the Shapiro-Wilk test, one-way analysis of variance (ANOVA) combined with the Tukey post-hoc test was employed to assess differences between groups. Statistical analysis was performed using GraphPad Prism software. ns: not significant, *P*≧0.05; *P<0.05; **P<0.01; ***P<0.001; ****P<0.0001.

Strikingly, rBCG-tMEV achieved breakthrough enhancement in Th1 responses. IFN-γ SFCs increased by 27.86% (*P* < 0.001), while IL-2 and TNF-α SFCs rose by 23.54% (*P* < 0.01) and 9.8% (*P* >0.05; ns), respectively, compared to BCG. Mechanistically, rBCG-tMEV induced synergistic effects via IL-2-mediated T cell proliferation and TNF-α-driven macrophage bactericidal activity, aligning with *Mtb* clearance mechanisms: IL-2 promotes antigen-specific T cell clonal expansion for long-term immune memory, whereas TNF-α stabilizes granulomas to limit pathogen dissemination.

BCG increased granzyme B (**Figure 10D**) and perforin (**Figure 10E**) SFCs by 146.93% and 171.05% (all *P* < 0.0001) versus tMEV. Compared with BCG, the secretion levels of granzyme B and perforin increased by 54.83% (*P*<0.001) and 32.03% (*P*<0.01), respectively, indicating that the secretory capacity of cytolytic molecules was enhanced.

Despite detectable IL-4 (**Figure 10F**) across groups, rBCG-tMEV showed no statistical difference in IL-4 SFCs compared to BCG (*P* > 0.05; ns).

#### 3.2.5 Protection experiment

To evaluate vaccine efficacy against *Mtb* dissemination, bacterial burden in lungs and spleens was quantified 8 weeks post-infection through colony-forming unit (CFU) enumeration (**Figure 11**). Both BCG and rBCG-tMEV immunization groups exhibited marked reductions in pulmonary and splenic CFU counts compared to PBS controls (*P* < 0.05). Notably, Compared with the phosphate-buffered saline (PBS) control group, the colony-forming unit counts in the lungs and spleens were significantly decreased in both the Bacillus Calmette-Guérin (BCG) group and the recombinant BCG-tMEV (rBCG-tMEV) immunization group (*P*<0.05). rBCG-tMEV immunization achieved the lowest mycobacterial load across organs, demonstrating enhanced protective efficacy over conventional BCG in this murine TB model.

**Figure 11:**
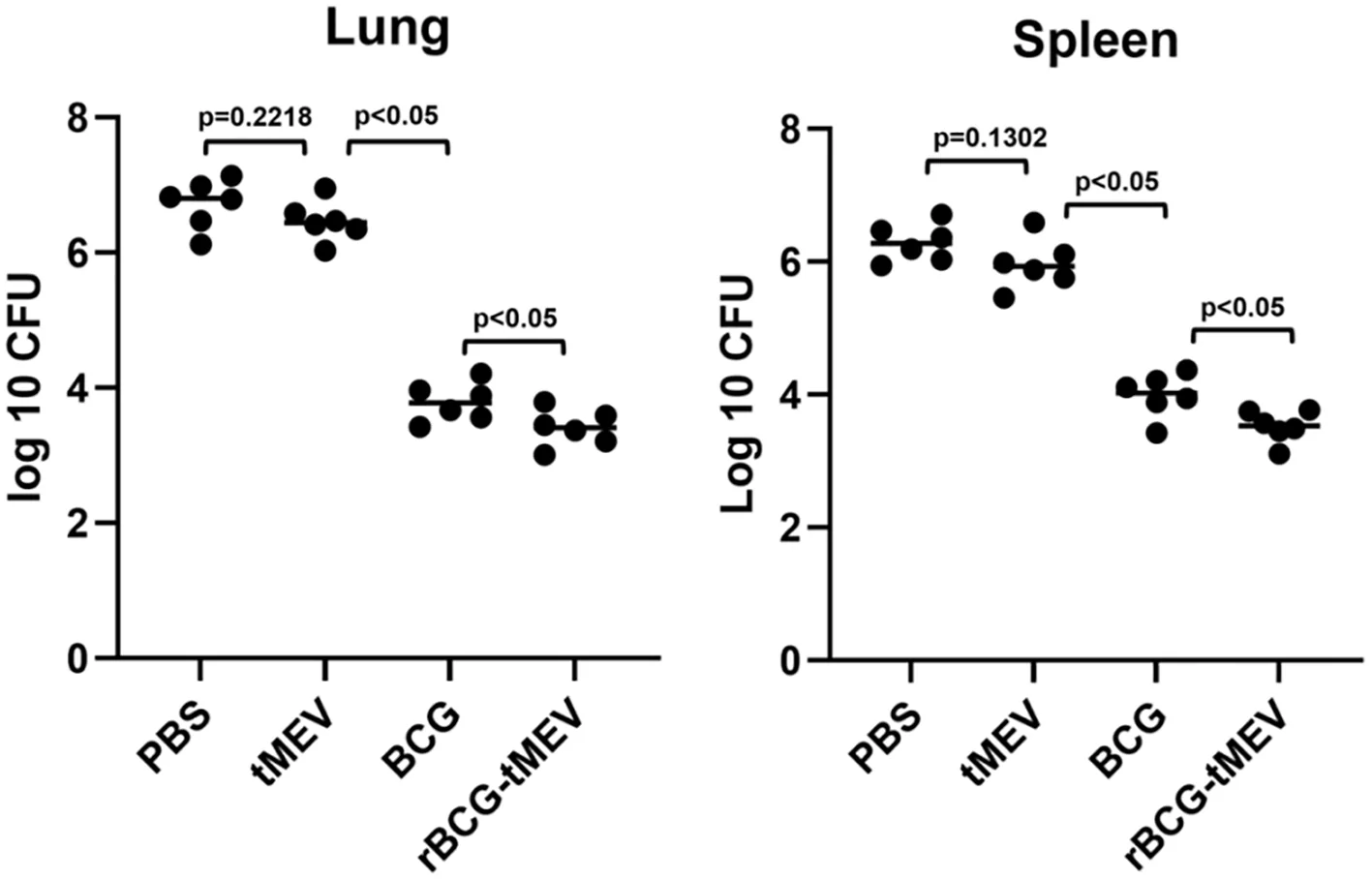
Vaccine efficacy against *Mtb* organ dissemination. (Left) Pulmonary mycobacterial load 8 weeks post-infection in vaccinated mice. (Right) Splenic mycobacterial load 8 weeks post-infection in vaccinated mice. Bacterial burden was quantified via colony-forming unit (CFU) enumeration. BCG and rBCG-tMEV immunization groups showed significant CFU reductions compared to PBS controls (*V* < 0.05). The data are expressed as mean ± SEM (n=6 per group). Following the verification of data normality using the Shapiro-Wilk test, a univariate analysis of variance (ANOVA), along with the Tukey multiple comparison method, was employed to assess the differences among the groups. The statistical analysis was conducted using GraphPad Prism.

## 4 Discussion

Despite the availability of standard drug treatment regimens, diagnostic methods, and vaccines, TB continues to pose a major threat to global health (Scriba et al., 2020). The development of new and effective vaccines is essential to the World Health Organization’s efforts to eliminate TB. Designing multi-epitope TB protein subunit vaccines using bioinformatics, structural information, and AlphaFold2 predictive models represents a promising strategy for inducing robust and durable immune responses (Kwon et al., 2018; Jumper et al., 2021^),^. The purpose of this study was to develop a multi-epitope recombinant BCG vaccine targeting Mtb, with the aim of enhancing immune responses in individuals infected with TB and preventing subsequent Mtb infection.

Multi-epitope TB vaccines target specific Mtb proteins, thereby limiting the functional scope of the protein and the range of available antigenic targets (Crosskey et al., 2020). The virulence of Mtb is associated with proteins secreted into host cells through the type VII secretion system (T7SS), providing a promising avenue for future drug and vaccine development (Zriba et al., [[NoYear]]). The fully characterized ESX-1 system substantially influences host-cell interactions, whereas ESX-3 and ESX-5 are critical for nutrient acquisition required for mycobacterial survival. Proteins secreted by the ESX-1 system are pathogen-specific and therefore constitute suitable targets because of their exogenous nature to the host (Sengupta et al., 2023). Notably, the RD-1 region is absent from BCG; however, the highly potent ESAT-6 protein secreted by ESX-1 can elicit strong cytotoxic T lymphocyte (CTL) responses against TB. If ESAT-6 is considered as a candidate vaccine protein, potential toxicity concerns must be thoroughly addressed (Kwon et al., 2018). In the development of multi-epitope vaccines, priority is given to the dominant peptide ESAT-6, and toxicity assessment is performed to select non-toxic T-cell and B-cell epitopes capable of triggering immune responses. Similarly, PE35, a component of the RD-1 region, is regarded as an immunodominant antigen with strong potential for vaccine development. Loss of PPE38 adversely affects the PPE-MPTR and PE_PGRS protein families, which function as surface antigens of Mtb and other pathogenic mycobacteria, thereby providing feasible vaccine targets (Nain et al., 2020). On this basis, dominant epitopes of the four aforementioned proteins were screened to construct a multi-epitope recombinant BCG. This aims to synergistically induce cellular and humoral immune responses at different stages of infection and reduce the risk of pathogen immune evasion via antigenic variation. The multi-epitope design can cover multiple supertypes including HLA-A, HLA-B and HLA-DRB1, enabling effective antigen presentation of the vaccine in populations with diverse genetic backgrounds. Meanwhile, overexpression of multi-epitope chimeric antigens allows the addition of immune adjuvants or targeted immune stimulation based on the adjuvant effect of the BCG vector, which can theoretically enhance and prolong protective efficacy. Early prediction of the physicochemical, antigenic, and allergenic properties of ESAT-6, PE35, PE4, and PPE38 showed that these proteins possess good stability, antigenicity, and non-allergenicity. CTLEs, HTLEs, LBEs, and CBEs from these proteins were subsequently predicted, enabling the selection of linked dominant epitopes for construction of the multi-epitope vaccine tMEV.

By analyzing and predicting the three-dimensional higher-order conformations of antigens, researchers can precisely optimize linear epitopes for T-cell receptors and conformational epitopes for B-cell receptors, thereby improving vaccine protective efficacy. Structural biology plays a central role in this process: atomic-level design precision enables epitopes to adopt immunogenically optimal native conformations, thereby eliciting potent and durable neutralizing antibody responses (Santiago, [[NoYear]]). This design principle has been validated in studies of foot-and-mouth disease virus, in which even subtle three-dimensional structural differences significantly affected neutralization efficacy, underscoring the critical importance of conformational fidelity for epitope function (Li et al., 2024). Moreover, such structure-guided precision design strategies are not only applicable to specific pathogens but also establish a general framework for multi-pathogen vaccine development. Therefore, systematic prediction, concatenation, and structural analysis of T/B-cell epitopes remain essential for optimal immunogen design. Using bioinformatics approaches, the secondary and tertiary structures of tMEV were predicted, and α-helices were identified as an ideal scaffold for exposing key epitope regions, accounting for 50.86% of the predicted structure. After optimization based on PROCHECK analysis, the three-dimensional structure of the model showed that approximately 98.4% of residues were located in favored regions and 1.6% in allowed regions, indicating excellent structural quality that meets the requirements for vaccine construction. It should also be noted that the structural prediction, molecular docking, molecular dynamics simulation and immune simulation presented in this study are computational methods for generating hypotheses and providing mechanistic insights. These in silico findings support, but do not replace, the experimental validation performed in this study. The immunogenicity and protective efficacy observed in the mouse model provide the primary experimental evidence for the potential of rBCG-tMEV as a vaccine candidate.

TLR4 is a transmembrane receptor located on the cytoplasm and vesicle membranes. During the immune response to *Mtb.* infection, it exerts multi-layered regulatory effects in pathogen recognition, innate immune cell activation, inflammatory response regulation, and adaptive immune shaping (Zhou et al., 2025). Mtb interacts with TLR4 via a variety of proteins to activate multiple signaling pathways including NF-κB, MAPK, and PI3K-AKT-mTOR (Guo et al., 2019). Therefore, TLR4 was selected as the interaction site for the tMEV protein. Molecular docking studies have shown that a stable and strong interaction between TLR4 and tMEV; subsequent molecular dynamics simulations further indicated well-defined and stable properties in terms of surface characteristics and interaction strength. The strong interaction between TLR4 and tMEV may enhance the anti-TB efficacy of tMEV.

The vaccine antigen tMEV was cloned into the plasmid pVM361 through optimized electroporation, followed by restriction enzyme digestion and ligation to ensure precise insertion. The recombinant plasmid was then transformed into *Mycobacterium bovis* BCG, and the recombinant strain rBCG-tMEV was obtained through selective culture. This approach leverages the intrinsic adjuvant properties of BCG while enabling sustained heterologous antigen expression—an essential feature for inducing durable T cell-mediated immunity against TB. As a multi-epitope vaccine delivery vector, Bacillus Calmette–Guérin (BCG) displays unique advantages in vaccine development by virtue of its outstanding safety profile, potent inherent immunoadjuvant activity and broad research and application prospects, rendering it a popular tool for vaccine research. On one hand, BCG can directly trigger dendritic cell (DC) maturation independent of the TNF-α signaling pathway: it upregulates the expression of maturation markers including surface MHC class II molecules, CD80, CD86 and CD83, boosts IL-12 secretion, and markedly enhances the antigen-presenting function of DCs, thereby possessing a distinctive direct immune activation pathway (Kim et al., 1999). On the other hand, BCG elevates the activity of cathepsin S (CTSS), improves the efficiency of tumor-associated antigen processing and presentation by DCs and macrophages, and further stimulates the activation of abundant melanoma-specific effector CD8^+^ T cells (Kremenovic et al., 2022).Advances in synthetic biology may further improve antigen expression efficiency (Wang et al., 2025) ^(^Madruga et al., 2026^),^ and accelerate clinical translation.

Traditional BCG vaccination regimens generally involve a single dose only. In recent years, scientists have paid growing attention to revaccination strategies to boost or sustain the immune protection elicited by BCG, with one core objective being the induction of long-lasting, antigen-specific T cell immune responses. Studies have suggested that effective booster vaccines can facilitate Th1 immune responses, augment the generation of memory T cells, and activate tissue-resident memory T cells, among other effects. Following HEHR vaccine boosting, sustained CD4+IFN-γ+IL-17A+ T cells (exhibiting the CXCR3+KLRG1- phenotype) were induced in the lungs of mice and remained detectable 12 weeks post-challenge (Kwon et al., 2024). The L91 booster vaccine significantly expands CD44hiCD62LhiCD127hi central memory CD4 T cells and CD44hiCD62LloCD127lo effector memory CD4 T cells, alongside increased polyfunctional Th1 cells (IFN-γ+TNF-α+) and Th17 cells (IFN-γ+IL-17A+) (Rai et al., 2017). Pulmonary-delivered peptide nanofiber vaccines expand the pool of Trm cells in lung tissue of BCG-primed mice, which is critical for local pulmonary immune defense (Files et al., 2022). The prime-boost vaccination regimen adopted in this study is similarly designed to maximize antigen exposure, amplify memory immune responses, and improve vaccine protective efficacy. Meanwhile, primary immunization delivers sufficient antigen or live bacteria to stimulate naive immune cells and lay the foundation for immune memory. A low antigen dose administered during boosting is sufficient to reactivate memory cells and markedly amplify antigen-specific immune responses. Additionally, a low live bacterial dose (10³ cfu) avoids excessive inflammation and organ damage in mice induced by high-dose BCG, striking a balance between immune potency and animal safety.

This study adopted a strategy combining immune simulation with *in vivo* serological and cytological assays to systematically evaluate the immunogenicity of rBCG-tMEV from two dimensions: mechanistic prediction and experimental validation. Immune simulation results indicated that upon antigen exposure, macrophages and dendritic cells rapidly internalize antigens and enter an antigen-presenting state, which subsequently drives marked clonal expansion and activation of helper T cells, accompanied by B cell activation, proliferation, and class switching from IgM to IgG1/IgG2, as well as the formation of long-lived memory T and B cell pools. The dominant secretion of IFN-γ alongside the concurrent elevation of proinflammatory cytokines including TNF-α and IL-6 in the cytokine profile further suggested a Th1-biased inflammatory microenvironment at the computational level. The complete kinetics of innate immune initiation, T cell activation and humoral immunity establishment predicted by the above simulations were supported by *in vivo* experiments. Specifically, ELISA analysis of human serum showed that antibodies in the serum of tuberculosis patients could recognize rBCG-tMEV. Furthermore, serological ELISA assays using murine serum yielded distinct immune polarization profiles among vaccine groups. The tMEV immunogen elicited a predominantly Th2-biased immune response, while parental BCG vaccination triggered a Th1-skewed immune phenotype. Notably, rBCG-tMEV immunization drove a balanced mixed Th1/Th2 immune response. The results of flow cytometry analysis showed that both CD4^+^ and CD8^+^ T-cell subsets exhibited significant expansion after immunization with rBCG-tMEV. ELISPOT assays showed simultaneous upregulation of Th1-associated IFN-γ and Th2-associated IL-4, indicating dual Th1/Th2 polarization. This phenomenon may arise from preferential activation of the MHC class II pathway through endocytosis of recombinant antigens, thereby promoting CD4^+^ T-cell differentiation into Th1 and Th2 effector subsets. Notably, immunization with tMEV antigen alone led to a significant increase in the proportion of CD4^+^ T cells and enhanced IL-4 secretion, further suggesting a Th2-biased response induced by exogenous linear epitopes. In pulmonary immune responses, a purely strong Th1 response may cause severe lung pathological injury, whereas appropriate Th2 responses or specific immunoregulatory mechanisms may help alleviate excessive inflammation (Verma et al., 2024). These results indicate that the recombinant rBCG-tMEV constructed in this study is expected to maintain efficient Th1-mediated bacterial clearance, and may balance pulmonary immunopathological damage through mixed immune responses induced by multiple antigen epitopes, though the specific mechanism remains to be verified in subsequent functional experiments.

In TB vaccine development, multiparametric analysis of cellular immune biomarkers is essential for comprehensive efficacy evaluation. Th1-polarized cytokines, particularly interferon-γ (IFN-γ), interleukin-2 (IL-2), and tumor necrosis factor-α (TNF-α), constitute key correlates of protection. TNF-α is also critically important for host control of Mtb, mediating phagocyte activation and granuloma organization (Ye et al., 2021). Notably, the cytolytic molecules granzyme B and perforin serve as functional surrogates of cytotoxic T lymphocytes (CTLs), whose pathogen-specific killing capacity via the granule exocytosis pathway is particularly important for controlling mycobacterial dissemination (Wang et al., 2023; Mandal et al., 2024). This integrated assessment strategy enables precise quantification of helper T cell-mediated immune regulation through Th1 cytokine profiling and assesses cytolytic immune responses via detection of cytolytic markers, providing a multidimensional framework for rational vaccine design and optimization. In this study, ELISPOT was used to systematically evaluate the immune characteristics of the recombinant rBCG-tMEV vaccine. The results indicated that rBCG-tMEV significantly enhanced Th1-type cellular immune responses (IFN-γ, IL-2, and TNF-α) and cytolytic-associated immune response (granzyme B and perforin), with superior efficacy compared with conventional BCG. For example, rBCG-tMEV induced IFN-γ and IL-2 secretion levels that were 27.86% and 23.54% higher than those induced by BCG, respectively; the release of CTL-associated effector molecules granzyme B and perforin increased by 54.83% and 32.03%, respectively. These results suggest that the recombinant vaccine may more effectively eliminate intracellular pathogens such as Mtb through synergistic activation of Th1 and CTL pathways. Flow cytometry further confirmed that rBCG-tMEV significantly expanded CD4^+^ and CD8^+^ T-cell populations. Expansion of CD8^+^ T cells is particularly critical because this subset can directly eliminate infected host cells through the granzyme–perforin pathway, thereby effectively restricting early mycobacterial dissemination (Irvine et al., 2021; Zhang et al., 2023), consistent with the experimental findings of this study.

Previous recombinant BCG (rBCG) vaccines (rBCG30, rBCG-AE, rBCG-ECD003) all overexpress full-length native proteins and have the following limitations: full-length proteins contain a large number of non-protective or immunodominance-shifted epitopes, which may induce ineffective or even regulatory immune responses. Furthermore, a study on the heterologous overexpression of Mycobacterium tuberculosis-derived Ag85B (Rv1886c) and PstS-1 (Rv0934) antigens in Mycobacterium smegmatis revealed significant differences in the proteomic and glycoproteomic profiles between the two recombinant strains and the wild-type strain, indicating that large proteins suffer from restricted intracellular expression and secretion efficiency in BCG (García-Ruiz et al., 2022). This study adopts a multi-epitope chimeric strategy, in which only dominant T-cell and B-cell epitopes are screened and concatenated while redundant sequences are eliminated, focusing antigens on the minimal functional units associated with immune protection, which can theoretically improve the immunogenic efficiency per unit antigen. PE/PPE family proteins were introduced during vaccine construction in this study. The inclusion of the PPE family antigen (Rv3619c) in the clinically advanced ID93 vaccine has been proven to enhance protective efficacy (Bertholet et al., 2010), indirectly supporting the rationality of the antigen selection strategy in this study. Vaccine designs based on Ag85A/Ag85B/ESAT-6/CFP-10 have suggested favorable immunogenicity and feasibility in silico (Nugraha et al., 2024). However, on the basis of bioinformatic analysis, this study further conducts comprehensive *in vitro* and *in vivo* experiments to verify the immunogenicity and feasibility of the recombinant vaccine from both in silico prediction and experimental validation dimensions, substantially improving the translational value and reliability of the research.

This study has certain limitations: during the epitope prediction stage, ELISA-based immunogenicity testing of individual candidate peptides was not performed prior to direct selection of highly reactive epitopes for linear concatenation. Although this strategy may represent a potential optimization approach for tMEV, further validation of immunogenic synergy and conformational stability is still required.

## 5 Conclusion

We constructed a multi-epitope vaccine tMEV and recombinant strain rBCG-tMEV by fusing immunodominant epitopes derived from ESAT-6, PE35, PE4 and PPE38 into the BCG backbone. In silico bioinformatic assessments indicated their robust antigenicity and predicted potent immunogenicity. *In vivo* murine experiments revealed that both candidates triggered balanced Th1/Th2 immune responses, markedly decreased mycobacterial burdens in the lung and spleen, and displayed satisfactory biosafety. Unadjuvanted standalone tMEV showed limited protective potency; therefore, we generated rBCG-tMEV via electroporation to deliver tMEV within BCG. Relative to parental BCG, rBCG-tMEV elicited synergistically augmented cellular and humoral immunity, overcoming the inadequate protection conferred by traditional BCG in adults. Overall, *in-vitro* and *in-vivo* experimental evidence confirms that tMEV and rBCG-tMEV show promising prospects as pre-clinical vaccine candidates. Further evaluation of their immunogenic efficacy and safety can be performed in models such as guinea pigs and non-human primates in the future. This dual-vaccine strategy is expected to boost protective effects against Mycobacterium tuberculosis and establish a research framework for the development of next-generation tuberculosis vaccines.

## Data Availability

The original contributions presented in this study are included in the article or **Supplementary Material**. For further inquiries, please contact the corresponding authors.
