# Supplementary material for: Immunogenicity and protective efficacy of recombinant BCG expressing a multi-epitope vaccine against mycobacterium tuberculosis
Source: Front Cell Infect Microbiol. 2026 Sep 30;16:1912983. doi: 10.3389/fcimb.2026.1912983 (PMC13661456; doi:10.3389/fcimb.2026.1912983)
Supplement: Supplementary file 1 [file DataSheet1.docx]

**Fig-S1**

**a**

**Figure S1.** Bioinformatics analysis of ESAT-6 (Rv3875), PE35 (Rv3872), PE4 (Rv0285), and PPE38 (Rv2352c). Transmembrane helix analysis **(a)** and signal peptide analysis **(b)** shows absence of transmembrane regions and signal peptides from all four vaccine candidates.

**Fig-S2**

**Figure S2. Molecular docking analysis of T cell epitopes with high-frequency MHC alleles.**

(a) Cytotoxic T lymphocyte (CTL) epitope interaction with HLA-A24:02, displaying a docking score of -218.00 (confidence score: 0.7958) and ligand RMSD of 115.23 Å. **(b)** Helper T lymphocyte (HTL) epitope binding to HLA-DRB115:01, showing enhanced affinity with a docking score of -252.10 (confidence score: 0.8851) and ligand RMSD of 116.51 Å. Negative docking scores indicate strong binding energy, while high confidence scores (>0.7) validate predictive reliability. RMSD values reflect structural stability during epitope-MHC complex formation.

**Fig-S3**

**Figure S3. Global population coverage analysis of prioritized T-cell epitopes.**

(a) Class I: MHC-I restricted epitopes. **(b)** Class II：MHC-II restricted epitopes. **(c)** Class combined: MHC-I restricted epitopes and MHC-II restricted epitopes.

**Fig-S4**

**Figure S4. Structural visualization of validated B-cell conformational epitopes in ESAT-6 protein:**

(a) ESAT-6: NIRQAGVQYS; **(b)** ESAT-6: AAWGGSGSEAYQ; **(c)** ESAT-6: VQYSRADEEQ; **(d)** ESAT-6: MAEMKTDAAT; **(e)** ESAT-6: STAGSLQGQWRGAAGTAAQAAVVRFQE; **(f)** ESAT-6: AAWGGSGSEAYQGVQQ.

**Note:** Only epitopes in the ESAT-6 protein passed the multi-criteria screening. All parameters (structural stability, hydrophilicity, antigenicity, non-allergenicity, non-toxicity) maintain consistency with T-cell epitope selection criteria, using identical evaluation thresholds

**Fig-S5**

**Figure S5. Validation of sequence-based solubility prediction model**

**(a)** "Scatter plot comparing experimental solubility (%) versus computational predictions (%). The red dashed line indicates the optimal classification threshold at 58% solubility, with the gray band representing the population average benchmark (PopAvrSol = 0.45). Data points include three independent datasets: Escherichia coli proteome (n=679), crystallization propensity test set (n=200), and UniProt-derived sequences (n=1,294), showing a Pearson correlation coefficient (r) of 0.621. Color gradient corresponds to QuerySol normalized values, where scores >0.45 (warmer colors) indicate above-average solubility relative to experimental observations."

**(b)** Surface physicochemical property mapping. "Electrostatic potential and hydrophobicity distribution visualized on protein structure using protein-sol patches algorithm. Blue regions: positive charge clusters (>+0.5 kT·e⁻¹), red areas: negative charge concentrations (<-0.5 kT·e⁻¹), yellow patches: hydrophobic domains (Wimley-White interfacial scale >3.0). Color scales were dynamically normalized based on the input structure's calculated electrostatic potential range."

**Fig-S6**

**Figure S6. Secondary structure prediction of the Epitope Vaccine (tMEV).**

(a) SOPMA analysis and **(b)** PSIPRED 4.0 prediction of tMEV secondary structure components, showing 50.86% α-helices, 6.47% β-turns, 36.85% random coils, and 5.82% extended strands. The high α-helical content supports structural stability, while the substantial proportion of random coils suggests flexible regions potentially involved in epitope accessibility. Notably, these secondary structure proportions align closely with those derived from the validated tertiary structure , confirming consistency across hierarchical structural predictions. The convergence of results from two independent prediction tools (SOPMA and PSIPRED 4.0) strengthens confidence in the tMEV's folding architecture.
